# Investigation and verification of the clinical significance and perspective of natural killer group 2 member D ligands in colon adenocarcinoma

**DOI:** 10.18632/aging.202935

**Published:** 2021-04-27

**Authors:** Guo-Tian Ruan, Shuai Wang, Li-Chen Zhu, Xi-Wen Liao, Xiang-Kun Wang, Cun Liao, Ling Yan, Hai-Lun Xie, Yi-Zhen Gong, Jia-Liang Gan, Feng Gao

**Affiliations:** 1 Department of Colorectal and Anal Surgery, The First Affiliated Hospital of Guangxi Medical University, Nanning, Guangxi Zhuang Autonomous Region, People's Republic of China; 2 Department of Immunology, School of Preclinical Medicine, Guangxi Medical University, Nanning, Guangxi Zhuang Autonomous Region, People's Republic of China; 3 Department of Hepatobiliary Surgery, The First Affiliated Hospital of Guangxi Medical University, Nanning, Guangxi Zhuang Autonomous Region, People's Republic of China; 4 Department of Thoracic Surgery, Affiliated Hospital of Guilin Medical College, Guilin, Guangxi Zhuang Autonomous Region, People's Republic of China; 5 Department of Gastrointestinal Surgery, Affiliated Hospital of Guilin Medical College, Guilin, Guangxi Zhuang Autonomous Region, People's Republic of China

**Keywords:** natural killer group 2 member D ligands, verification, colon adenocarcinoma, diagnosis, prognosis

## Abstract

This study investigated and verified the diagnostic and prognostic values of natural killer group 2 member D ligand (NKG2DL) genes in colon adenocarcinoma (COAD). We downloaded *NKG2DL*s expression data and corresponding clinical parameters from The Cancer Genome Atlas (TCGA) and used bioinformatics techniques to investigate the values of *NKG2DL*s in COAD. Then, we used the GSE40967 cohort to verify the prognostic value of *NKG2DL*s. Finally, we verified the ULBP2 expression level in tissues, and also investigated the diagnostic and prognostic values of ULBP2 in COAD. The diagnostic receiver operating characteristic curves showed that *ULBP1*, *ULBP2*, *ULBP3,* and *RAET1L* had high diagnostic values in COAD [Area Under Curve (AUC) > 0.9]. In TCGA cohort, the univariate and multivariate survival analyses suggested that *ULBP2* was correlated with the prognosis of COAD recurrence-free survival (RFS) and overall survival (OS). In GSE40967 cohort, *ULBP2* was associated with CC RFS and OS. Reverse transcription-quantitative polymerase chain reaction and immunohistochemistry results showed that ULBP2 was highly expressed in COAD tumor tissues (*P* < 0.05) and both had diagnostic values (AUC > 0.7). Validated survival analysis showed that the high expression of ULBP2 had a worse prognosis in COAD OS and RFS. Thus, *ULBP2* might be an independent diagnostic and prognostic biomarker of COAD.

## INTRODUCTION

Colorectal cancer (CRC) is one of the most alarming malignant tumors or cancers in the world, and its morbidity and mortality have always been key issues of concern, globally. According to the 2018 GLOBOCAN global cancer data, more than 1.8 million people were diagnosed with CRC in 2018, while the number of the deaths related to CRC was 881,000, ranking third in terms of global morbidity and second in mortality [1]. The American Cancer Society evaluated newly occurred cancer cases and deaths in the United States in 2020 and showed that the incidence and mortality of CRC ranked third in malignant tumors, in both men and women [2]. In China, the incidence and mortality of CRC are on the rise [3]. Histologically, CRC can be classified as adenocarcinoma, adenosquamous carcinoma and undifferentiated carcinoma. Colon adenocarcinoma (COAD) is the most common histological subtype. [4] In recent years, the incidence and mortality of COAD have shown a significant upward trend, especially in Western developed countries and Asian developing countries [5, 6]. At present, the treatment of colon adenocarcinoma is comprehensive, including radiotherapy, chemotherapy and surgery, but the five-year survival rate of COAD is still not ideal [7]. Clinical predictors such as TNM (tumor-node-metastasis) stage, sex, and age represent conventional methods for evaluating prognosis, but these methods cannot accurately predict the survival of patients. Although serum microRNA has been used to evaluate early COAD, its diagnostic advantages are not very competitive compared with other non-invasive tests [8]. Colonoscopy is the gold standard for the diagnosis of COAD, but it has not been widely used due to its high cost and invasiveness [9, 10]. Serum carcinoembryonic antigen (CEA) is an important tumor marker for COAD diagnosis and postoperative monitoring, but in some clinical trials, the positive rate of serum CEA in patients is less than 50% [11, 12]. Therefore, it is of great value to identify and screen out accurate and practical biomarkers related to COAD diagnosis and prognosis.

Human intestinal intraepithelial lymphocytes (IELs), which are T cell receptor αβ^+^ CD8^+^ T cells located between epithelial cells (EC), may be involved in the innate immune response against colon cancer. They can detect or kill the stunted or malignant ECs [13]. NKG2D (natural killer group 2, member D) is a C-type lectin-like activation receptor expressed in NK cells and various T cell subsets including CD8^+^ cytotoxic T cells. [14] These molecules can identify the major histocompatibility complex (MHC) class I polypeptide-related sequence (MIC) A/B protein and unique long 16 (UL16)-binding protein (ULBP) on EC, collectively known as NKG2D ligands (NKG2DL) [15–18]. MICA/B has the same α1, α2, and α3 domains as MHC class I, and the α3 domain is an Ig-like domain [19]. The unique long 16 (UL16)-binding protein (*ULBP*) family, also known as retinoic acid early transcript (*RAET*), is a cell membrane protein family expressed on transformed and stressed cells [20, 21]. In humans, the *ULBP* family contains 6 functional members, including GPI anchored proteins [*ULBP1* (*RAET1I*), *ULBP2* (*RAET1H*), *ULBP3* (*RAET1N*), *ULBP6* (*RAET1L*)] and transmembrane proteins [*ULBP4* (*RAET1E*), *ULBP5* (*RAET1G*)] [22, 23]. Interestingly, ULBPs only have α1 and α2 domains [20]. Studies of cancer models *in vivo* strongly suggest that the activated immune receptor NKG2D is involved in the anti-cancer immune response [24, 25]. The NKG2DLs expressed on tumor cells and bound to the NKG2D receptor can activate NK cells to kill tumor cells. Additionally, when combined with the NKG2D receptor on T cells, they can provide costimulatory signals [16]. Some previous studies reported the decrease of NKG2D-positive NK cells in colorectal cancer (CC), gastric cancer (GC) and pancreatic cancer (PC) were associated with a poor prognosis [26]. NKG2DLs were rarely expressed in normal healthy tissues, but found in a variety of different cancer-derived cell lines and primary cancers [27]. However, to our knowledge, the relationship between the *NKG2DL* family genes and COAD has not been studied. Therefore, our study aimed to explore and discover the potential clinical values of *NKG2DL* family genes in COAD, particularly in its diagnostic and prognostic values.

## RESULTS

### Public database information mining

We downloaded the mRNA expression of the *NKG2DL* genes from a total of 456 patients diagnosed with COAD, including 480 tumor tissue samples and 41 adjacent normal tissue samples from the TCGA database. We excluded those patients with repetitive information and a survival time of 0, and we obtained 438 tumor samples and 41 adjacent tissue samples. We integrated the gene expression data with the corresponding clinical parameters downloaded from the TCGA database. The results are shown in Supplementary Table 1. In the RFS cohort, in addition to age and tumor location, other clinical parameters were associated with COAD RFS (all *P* < 0.05). As for the OS cohort, the majority of clinical parameters were associated with COAD OS (except for age, sex and tumor location, all *P* < 0.05). Finally, to validate the prognostic value of NKG2DL genes, we downloaded the prognostic information of 586 CC patients related to the expression of the *NKG2DL* family genes (with the exception of *ULBP5* (*RAET1G*) and *ULBP6* (*RAET1L*)) from the GEO database, we excluded those cases without survival information, leaving 580 cases of OS and 575 cases of RFS (Supplementary Table 2). In the RFS cohort, TNM stage, chemotherapy adjuvant, Kirsten rat sarcoma viral oncogene (KRAS) mutation and Cartes d'Identité des Tumeurs (CIT) molecular subtype were related to CC RFS (*P* < 0.05); in the OS cohort, age, TNM stage, KRAS mutation and CIT molecular subtype were related to CC OS (*P* < 0.05).

### Bioinformatics analysis

The GO and KEGG analyses suggested that *NKG2DL* genes might be involved in the process of natural killer cell mediated cytotoxicity (Supplementary Figure 1A). The GO analysis by BiNGO in Cytoscape found a similar result in natural killer cell activity (Supplementary Figure 1B–1D). The natural killer cell-mediated cytotoxicity pathway diagram showed that the *NKG2DL* genes were mainly involved in the regulation of the MAPK signaling pathway (Supplementary Figure 2). As for the association of *NKG2DL* genes, our Pearson’s correlation analysis showed that *MICA* and *MICB*, *ULBP2* and *ULBP3*, *ULBP2* and *RAET1L*, *ULBP3* and *RAET1L*, *RAET1E* and *RAET1G*, *RAET1G* and *RAET1L* had a specific relationship with each other (Supplementary Figure 1E). Finally, an investigation of the interaction networks of *NKG2DL* genes showed that MIC genes and *NKG2DL* members had solid homology and co-expression relationships (Supplementary Figure 1F–1G).

### Expression levels of *NKG2DL* genes in normal colon tissues and COAD tumor tissues

The expression of *NKG2DL* genes in normal human colon tissues was collected from the GTEx database (Supplementary Figure 3). The expression data of *NKG2DL* genes in tumor tissues from COAD patients and normal tissues were downloaded from the GEPIA website and showed that the *MICA* gene was lowly expressed in COAD tumor tissues, while *MICB*, *ULBP1*, *ULBP2*, *ULBP3*, *RAET1E*, *RAET1G,* and *RAET1L* were highly expressed in COAD tumor tissues (Supplementary Figure 4).

### Differential gene expression and diagnostic ROC curve analysis based on the TCGA cohort

**Figure 1:**
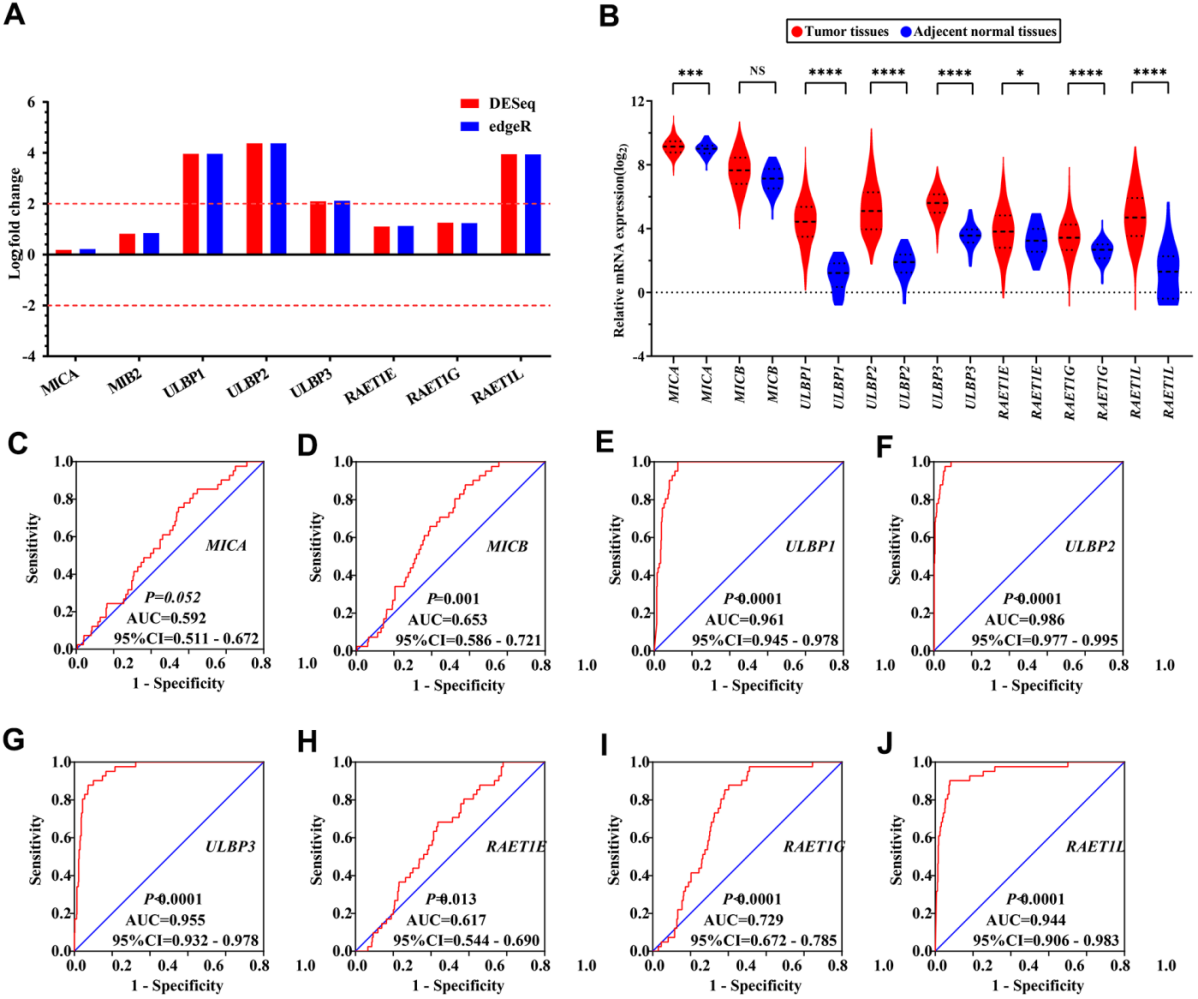
**The differential expression and diagnostic ROC curves of NKG2DL genes in COAD**. (**A**, **B**) The differential expression of NKG2DL genes in COAD: (**A**) Differential expression in the Edger and DESeq; (**B**) Expression distribution in TCGA; (**C**–**J**) The diagnostic ROC curves of NKG2DL genes in distinguish COAD tumor tissues and adjacent normal tissues in TCGA cohort: ROC curves of MICA (**C**); MICB (**D**); ULBP1 (**E**); ULBP2 (**F**); ULBP3 (**G**); RAET1E (**H**); RAET1G (**I**); RAET1L (**J**). Notes: COAD: colon adenocarcinoma; NKG2DL, Natural Killer Group 2 MemberD Ligand; ULBP: unique long 16 (UL16)-binding protein; MIC: Human Major Histocompatibility Complex (MHC) class I polypeptide-related sequence; TCGA: The Cancer Genome Atlas; NS: not significant; ROC: receiver operating characteristic; AUC: area under the curve; CI: confidence interval. * P<0.05; *** P<0.001; **** P<0.0001.

The results of differential expression analysis of *NKG2DL* genes in COAD showed that *ULBP1*, *ULBP2*, *ULBP3* and *RAET1L* had significant differential expression in COAD (all log2 fold change ≥ 2.0) (Figure 1A). This analysis suggested that all *NKG2DL* genes were highly and significantly expressed in tumor tissues, but their expression levels in adjacent normal tissues were low (Figure 1B). The diagnostic ROC curve analyses suggested that *ULBP1*, *ULBP2*, *ULBP3*, *RAET1G* and *RAET1L* had diagnostic value in COAD (AUC > 0.7), among them *ULBP1* [AUC (95% CI) = 0.961 (0.945-0.9780)], *ULBP2* [AUC (95% CI) = 0.986 (0.977–0.995)], *ULBP3* [AUC (95% CI) = 0.955 (0.932–0.9780) and *RAET1L* [AUC (95% CI) = 0.944 (0.906–0.983)] had higher diagnostic values (AUC > 0.9) (Figure 1C–1J).

### Survival analysis

**Figure 2:**
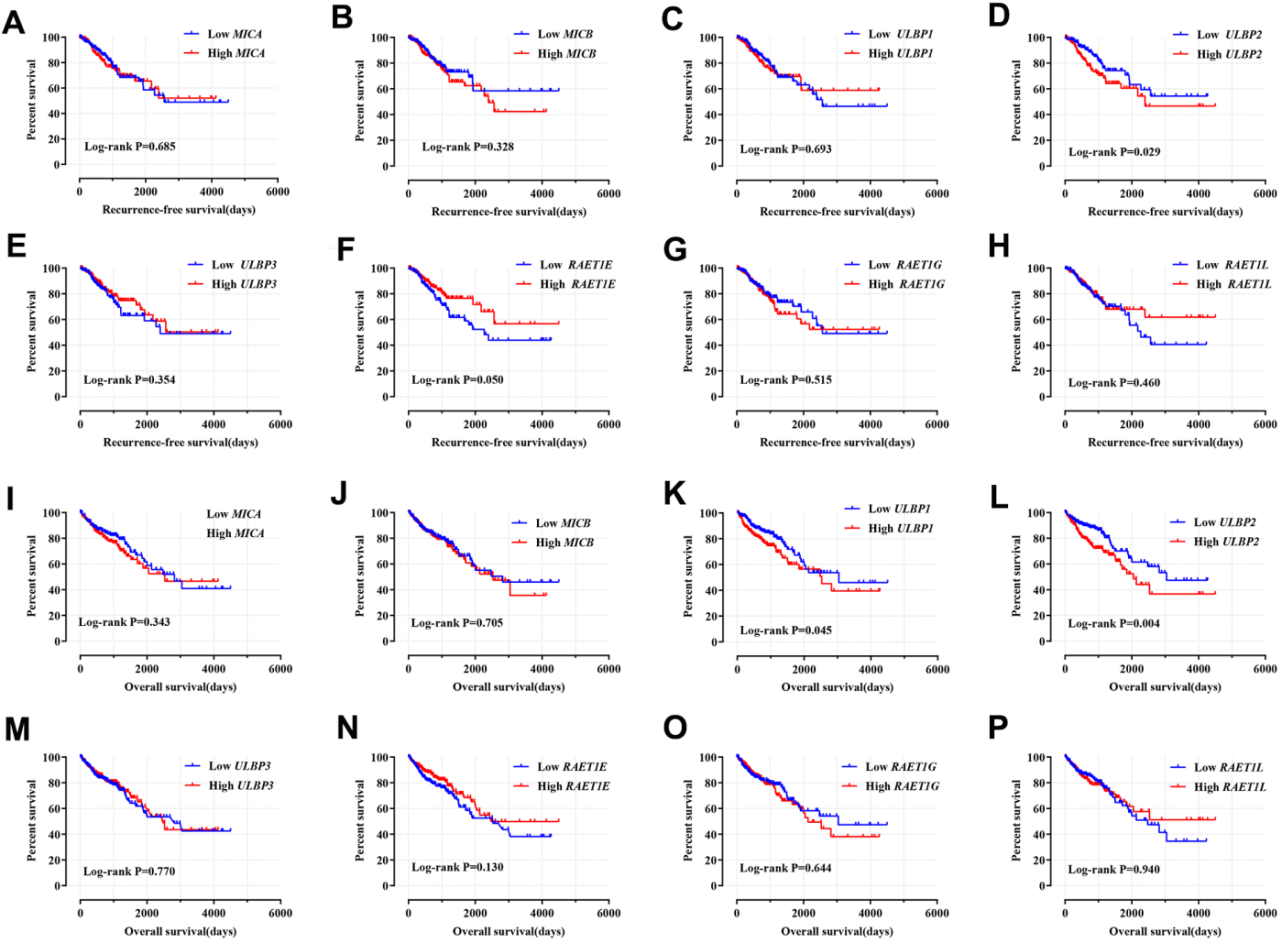
**Kaplan–Meier survival curves for *NKG2DL* genes in COAD of TCGA cohort.** Notes: RFS stratified by *MICA* (**A**); *MICB* (**B**); *ULBP1* (**C**); *ULBP2* (**D**); *ULBP3* (**E**); *RAET1E* (**F**); *RAET1G* (**G**); *RAET1L* (**H**). OS stratified by *MICA* (**I**); *MICB* (**J**); *ULBP1* (**K**); *ULBP2* (**L**); *ULBP3* (**M**); *RAET1E* (**N**); *RAET1G* (**O**); *RAET1L* (**P**). COAD: colon adenocarcinoma; *NKG2DL*, Natural Killer Group 2 Member D Ligand; *ULBP:* unique long 16 (UL16)-binding protein; *MIC*: Human Major Histocompatibility Complex (MHC) class I polypeptide-related sequence; TCGA: The Cancer Genome Atlas; RFS, recurrence-free survival; OS, overall survival.

First, based on the TCGA cohort, we performed prognostic survival analysis of the *NKG2DL* genes in COAD, the multivariate analyses showed that the high expression of *ULBP2* [adjusted *P* = 0.007, HR (95% CI) = 2.490 (1.349–6.405)] and *RAET1E* [adjusted *P* = 0.009, HR (95% CI) = 0.236 (0.157–0.764)] were correlated with the prognosis of COAD RFS. At the same time, the *ULBP2* gene was involved in the COAD OS [adjusted *P* = 0.025, HR (95% CI) = 1.765 (1.074–2.901)], and the low regulation of *ULBP2* had a favorable prognosis of COAD OS (Figure 2, Supplementary Table 3). In addition, the GSE40967 cohort based on the GEO database was used to validate the prognostic values of *NKG2DL*, the results suggested that the expression of the *ULBP2* gene [adjusted *P* = 0.036, HR (95% CI) = 1.423 (1.024–1.979)] was related to the RFS of CC; meanwhile, the expression of the *MICB* gene (adjusted *P* = 0.037, HR (95% CI) = 0.722 (0.532)–0.980)] and *ULBP2* gene [adjusted *P* = 0.005, HR (95% CI) = 1.563 (1.146–2.130)] were related to the OS of CC (Supplementary Figure 5, Supplementary Table 4). The cohort comparison between two databases found that *ULBP2* gene was related to the RFS and OS of COAD and CC.

### Comprehensive prognosis survival analysis

**Figure 3:**
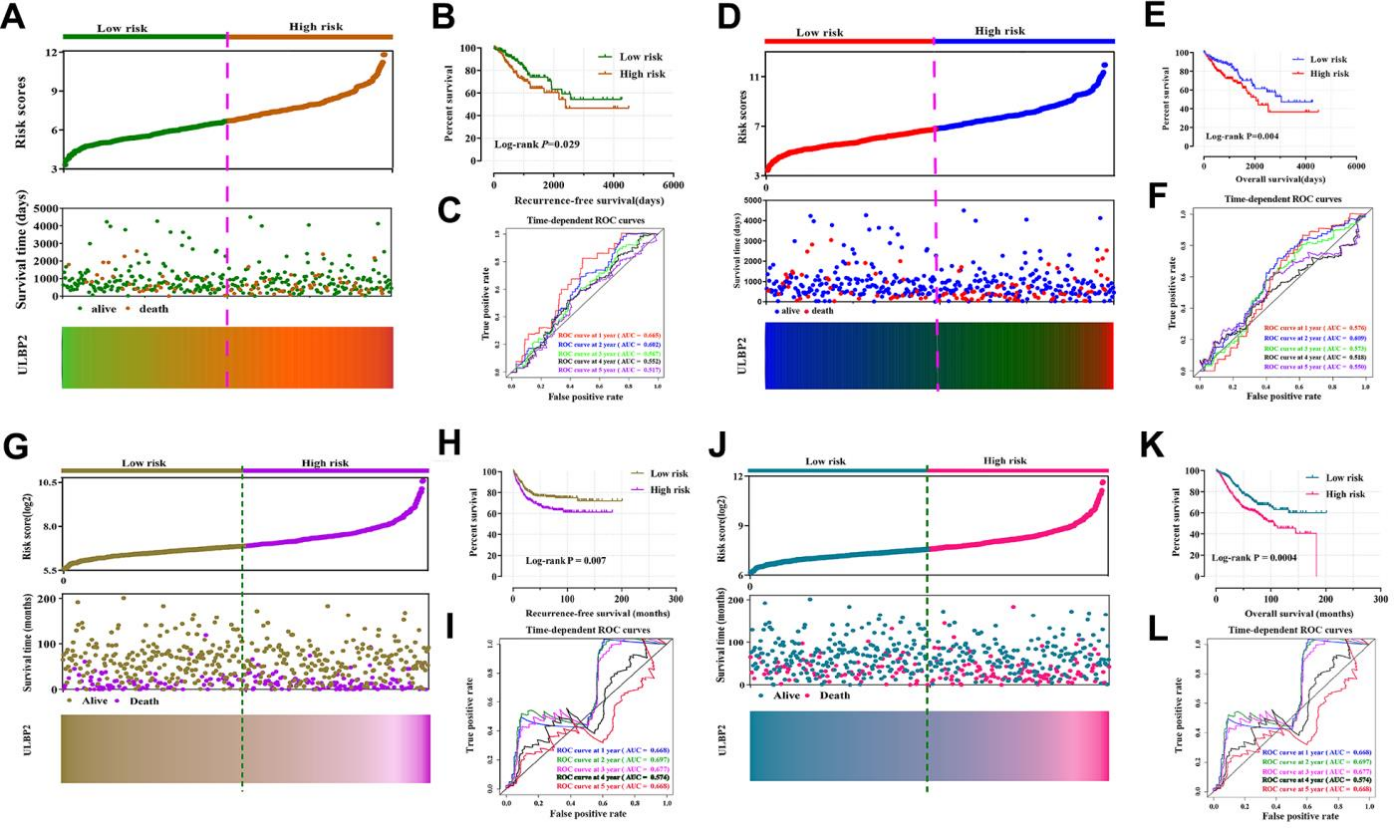
**Prognostic risk score model and time-dependent ROC curve of *ULBP2* gene in COAD and CC RFS and OS based on TCGA cohort and GSE40967 cohort**. (**A**, **D**) Risk score model plot including risk score ranking, survival status, and heatmaps in COAD in COAD RFS and OS; (**B**, **E**) Kaplan–Meier curves for low- and high-risk groups in COAD RFS and OS; (**C**, **F**) ROC curves for 1-, 2-, 3-, 4-, and 5-year survival rates from the risk score model in CC RFS and OS; (**G** and **J**) Risk score model plot including risk score ranking, survival status, and heatmaps in CC in CC RFS and OS; (**H**, **K**) Kaplan–Meier curves for low- and high-risk groups in CC RFS and OS; (**I**, **L**) ROC curves for 1-, 2-, 3-, 4-, and 5-year survival rates from the risk score model in CC RFS and OS. Notes: COAD: colon adenocarcinoma; CC: colon cancer; *ULBP:* unique long 16 (UL16)-binding protein; ROC, receiver operating characteristic; RFS, recurrence-free survival; OS, overall survival.

We performed a comprehensive analysis of the prognostic value of *ULBP2* in RFS and OS of COAD and CC. Nomogram results showed that the *ULBP2* gene had a certain contribution to the RFS and OS of COAD and CC (Supplementary Figure 6). However, it was also found that the TNM stage contributed the most to the risk score. Next, we investigated the association of the TNM stage with *ULBP2* gene which we downloaded from the GEPIA website, and the result indicated that the TNM stage had no connection with *ULBP2* (*P* > 0.05) (Supplementary Figure 7). Finally, we constructed a prognostic risk model of *ULBP2* gene for COAD and CC. The higher the expression of the *ULBP2* gene, the higher the prognostic risk score and the greater the mortality rate. In the TCGA cohort, the low-risk model group had better prognostic value for RFS and OS (log-rank *P* = 0.029 and log-rank *P* = 0.004), the 1-, 2-, 3-, 4, and 5- year survival ROC curves showed that the AUC values were about 0.5–0.6 (Figure 3A–3F). In the GEO cohort, it was found that the high-risk model group had a worse prognosis for RFS and OS (Log-rank *P* = 0.029 and log-rank *P* = 0.004), the 1-, 2-, 3-, 4, and 5- year survival ROC curves showed that the AUC values were about 0.5–0.6 (Figure 3G–3L).

### Verification of ULBP2 expression in COAD tumor tissues and adjacent normal tissues

### RT-qPCR of ULBP2 expression of COAD in the Guangxi cohort

**Figure 4:**
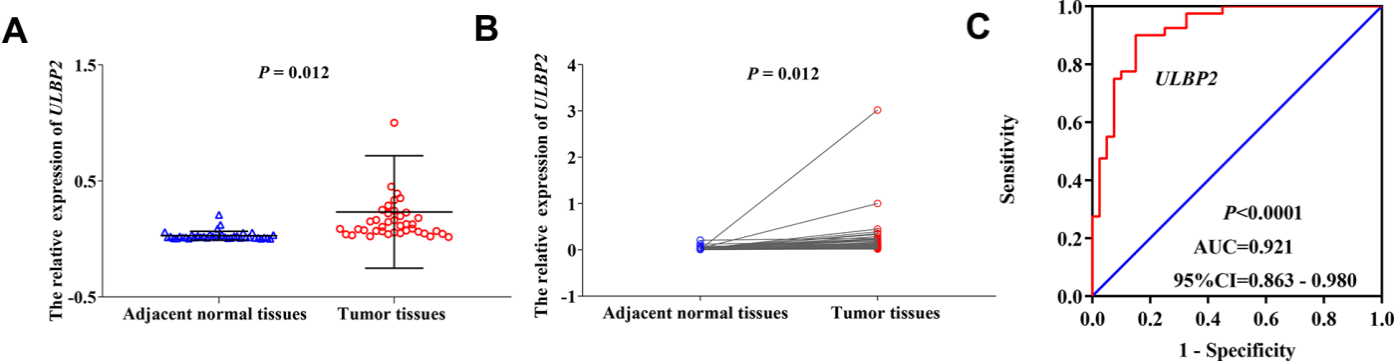
**The relative expression and diagnostic ROC curve of ULBP2 gene in COAD tumor tissues and adjacent normal tissues**. Notes: (**A**, **B**) The relative expression of ULBP2 gene; (**C**) diagnostic ROC curve. Notes: COAD: colon adenocarcinoma; ULBP: unique long 16 (UL16)-binding protein; AUC: area under the curve; CI: confidence interval.

We used RT-qPCR technology to detect the relative expression of the *ULBP2* gene in COAD tumor tissues and adjacent normal tissues, and the results indicated that the relative expression was significantly higher in COAD tumor tissues (0.23233246±0.48443959501) than in adjacent normal tissues (0.02814684 ± 0.037491460) (*P* = 0.012; Figure 4A, 4B). Besides, we performed the diagnostic ROC curve to evaluate the diagnostic value of the *ULBP2* gene in COAD. The results suggested that the *ULBP2* gene had a higher diagnostic value in COAD (*P* < 0.0001, AUC = 0.921, 95% CI = 0.863–0.980; Figure 4C).

### IHC of ULBP2 expressions of COAD in Guangxi cohort

**Figure 5:**
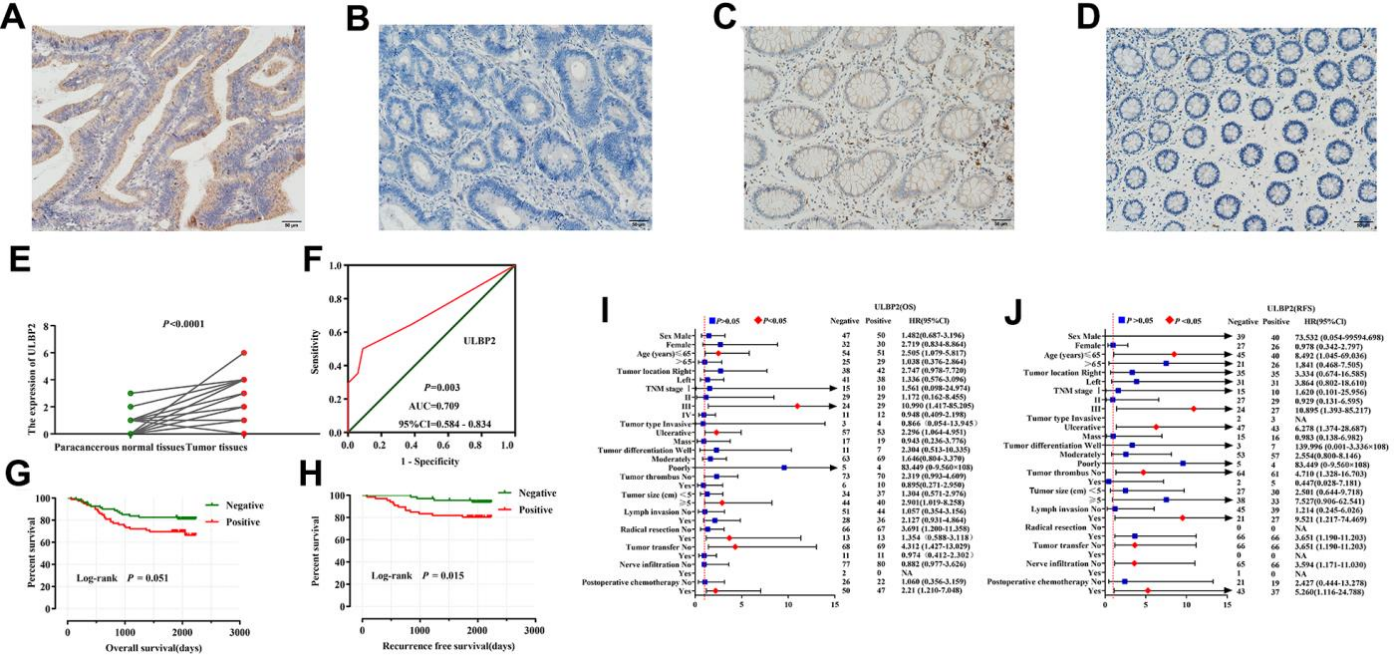
**Immunohistochemical staining analyses of ULBP2 expression in COAD tumor tissues and adjacent normal tissues**. (**A**–**D**) ULBP2 staining results were observed in the cytoplasm of colon cells; (**A**) Positive staining of COAD tumor tissues; (**B**) Negative staining of COAD tumor tissues; (**C**) Positive staining of adjacent normal colon tissues; (**D**) Negative staining of adjacent normal tissues; (**E**, **F**) Diagnostic analysis of ULBP2 expression in COAD: (**E**) Scatter plot; (**F**) Diagnostic ROC curve; survival analysis of ULBP2 expression in COAD: (**G**, **H**) Kaplan–Meier survival curve of ULBP2 protein expression in COAD OS and RFS; (**I**, **J**) Stratified analysis of ULBP2 expression in different clinical parameter layers in COAD patients: (**A**) OS; (**B**) RFS. Notes: COAD, colon adenocarcinoma; *ULBP:* unique long 16 (UL16)-binding protein; ROC, receiver operating characteristic; AUC: area under the curve; CI: confidence interval; OS, overall survival; RFS, recurrence-free survival. Magnification, 200×.

The IHC results showed that ULBP2 was mainly expressed in the cytoplasm. ULBP2 protein expression was seen in 79 of 161 (49.1%) COAD patient tumor samples, while ULBP2 was only seen in 3 of 34 (8.8%) adjacent normal tissues (Figure 5A–5D).

The differential expression analysis showed that the IHC score of ULBP2 protein in COAD tumor tissues was significantly higher than in adjacent normal tissues (*P* < 0.0001), and the diagnostic ROC curve analysis suggested that the IHC score of ULBP2 protein has a certain diagnostic value in COAD [*P* = 0.003, AUC [95% CI =0.709 (0.584–0.834)] (Figure 5E, 5F).

Our prognostic survival curves showed that patients with negative expression of ULBP2 protein in COAD had a better OS and RFS (Figure 5G, 5H), and the results of Multivariate COX regression analysis showed that ULBP2 protein expression was related to COAD OS [adjusted *P*=0.047, HR (95% CI) =2.009 (1.009–4.001)], while ULBP2 protein expression was related to COAD RFS [adjusted *P*=0.032, HR (95% CI) =9.521 (1.217–74.469)] (Supplementary Table 5).

The stratified analysis results showed that young (≤ 65 years), TNM stage III, ulcerative COAD, tumor diameter (≥ 5cm), non-radical surgical resection, no tumor metastasis, and postoperative adjuvant chemotherapy patients with positive ULBP2 protein expression increased COAD patients risk of death and the OS was shorter; while young (≤ 65 years), TNM stage III, ulcerative COAD, tumor thrombosis, non-radical treatment surgical resection, no tumor metastasis and postoperative adjuvant chemotherapy patients with negative expression of ULBP2 protein decreased the risk of death in COAD patients, and the RFS was longer (Figure 5I, 5J).

## DISCUSSION

In the colonic intestinal epithelium, the immune system affects the occurrence and development of tumors. Clinical data suggest that immune surveillance plays an important role in inhibiting and killing tumor cells in a variety of human cancers, including colon cancer [28]. In several immune mechanisms, the NKG2D receptor-ligand system might play a leading role in protecting the host from cancer [29]. Research on cancer models *in vivo* showed strongly that the activated immune receptor NKG2D participated in the anti-cancer immune response [24, 30, 31]. It has been reported that deficiency of NKG2D could increase the incidence of malignancies in mice [32]. *NKG2DL* genes bound to the NKG2D receptor can activate NKG2D to support the cytotoxic activity of NK cells and T cells against tumor cells [33]. Tumor cells regulate the expression of *NKG2DL*s at the transcription, translation and post-translational levels, thereby evading the recognition of NK cells. Many previous studies have found that changes in *NKG2DL* gene expression could be related to the diagnosis and prognosis of cancer [16]. Therefore, this study aimed to investigate the diagnostic and prognostic values of *NKG2DL* genes in COAD.

In our study, we collected the expression mass spectra of *NKG2DL* family genes in human colon normal tissues from the GTEx database. It was seen that *MICB*, *ULBP1*, *ULBP2*, *ULBP3*, *RAET1E*, *RAET1G,* and *RAET1L* (except *MICA*) were expressed at relatively low levels in normal colon tissues. On the other hand, when we integrated the gene expression levels of the NKG2DL family collected from the GEPIA website and the TCGA database in COAD patient tumor tissues and normal tissues, the results also found that the expression levels of MICB, ULBP1, ULBP2, ULBP3, RAET1E, RAET1G and RAET1L (except *MICA*) were consistent. In our study, the MICA gene was highly expressed in the tumor tissues of COAD patients, which is consistent with some previous results [34–38].

It has been reported that genetic and epigenetic alterations could be involved in the tumorigenesis and development process of cancers (such as colon cancer) and that these modifications and alterations could expose tumor cells to the immune system [39–41]. Our differential expression analysis results showed that *ULBP1*, *ULBP2*, *ULBP3,* and *RAET1L* had significant differential expression in COAD tumor and adjacent normal tissues. We then performed a diagnostic ROC curve analysis of the *NKG2DL* family genes and found that *ULBP1*, *ULBP2*, *ULBP3* and *RAET1L* had high diagnostic values in COAD. A study by McGilvray et al. [42] on NKG2D ligands expression in a total of 462 primary colorectal tumors found that the majority of colorectal tumors expressed the NKG2D ligands. Kamei et al. [43] investigated the expression levels of *ULBP* genes and *NKG2D* in 98 patients who received surgery, human gastric cancer cell lines (MKN-74 cells) and gastric cancer tissues from 2004 to 2008, and they found high expression of *ULBP1* and ULBP2/5/6 on the MKN-74 cells surface, and that the *ULBP1* expression was positive in 70/98 cases. The same result was found in cholangiocarcinoma [44]. Kruijf et al. [45] studied 677 breast cancer patients from 1985 to 1994 to verify the expression of NKG2D ligands (NKG2DL) using immunohistochemical staining and they found that NKG2DL was frequently expressed by tumors (ULBP1, 90% of the cases; ULBP2, 99%; ULBP3, 100%; ULBP4, 26%; ULBP5, 90%).

The comprehensive analysis of prognostic values and diagnostic values of COAD based on the TCGA and GEO cohorts suggested that the *ULBP2* might be an independent diagnosis and prognosis indicator (both of RFS and OS) in COAD. Additionally, in our study, we verified the ULBP2 expression in cells and tissues, and we also analyzed the diagnostic and prognostic values of ULBP2 in COAD. Our results found that ULBP2 expression in COAD tumor tissues was higher than in adjacent normal tissues. The survival analysis results suggested that the positive expression of ULBP2 indicated a poor prognosis of COAD. The diagnostic and prognostic values of *ULBP2* in cancer were reported in a lot of previous studies. From the perspective of immunohistochemistry, Chang et al. [46] investigated the expression of *ULBP2* in pancreatic cancer (PC) and *ULBP2* serum levels in 154 early-stage PC patients and 142 healthy participants by means of an immunoassay. They found that *ULBP2* was expressed more highly in PC tissues than in adjacent normal tissues and that the diagnostic ROC curve had a diagnostic value in PC (AUC = 0.862). A similar result was reported in the Zhou et al. [47] study, in which the AUC was 0.923. Li et al. [48] used immunohistochemical staining to examine the expression of the *ULBP2* in tissues from 82 cases of ovarian cancer and 6 cases of tissues from patients without ovarian cancer. Their results showed that *ULBP2* was expressed in 82.9% (68/82) of the cases, but not expressed in any of the normal ovarian tissues. They also analyzed the prognosis of ovarian cancer patients. The Kaplan-Meier curve and log-rank test suggested that the overall (75 ± 12 months) and progression-free (53 ± 11 months) survival of patients with high expression of *ULBP2* was significantly worse than that of those with low expression of *ULBP2* (112 ± 8 months, 107 ± 9 months, respectively), *P* < 0.05. In terms of immunohistochemical staining, Tsukagoshi et al. [44] analyzed 82 cases of extrahepatic cholangiocarcinoma (EHCC) tissues to test the expression of NKG2D ligands, and they found that high expression of *ULBP2* was related overall and disease-free survival. Paschen et al. [41] used immunohistochemistry to examine the expression of *ULBP2* in melanoma tissues and soluble molecules in sera from > 200 melanoma patients. They discovered that *ULBP2* was highly expressed in melanoma tissues and that the high concentration of sULBP2 was significantly associated with disease progression, tumor load and reduction in overall survival. The prognostic value of *ULBP2* was also reported in B-cell chronic lymphocytic leukemia (CLL), and the expression in 98 patients with CLL and 48 healthy participants as the control group was analyzed by means of ELISA. It was found that the sULBP2 was highly expressed in CLL patients (sULBP2 > 105 pg/mL) and associated with poor treatment-free survival (TFS) [49]. Besides, the prognostic value of *ULBP2* was also found in ovarian cancer and breast cancer [21, 45]. Gao et al. [50] downloaded and analyzed the prognostic value of lncRNA, miRNA and mRNA genes in colon cancer patients from the TCGA database and found that ULBP2 is highly expressed in CC and the high expression of ULBP2 in CC indicated a poor prognosis for CC [51]. When Wang et al. collected and analyzed the mRNA datasets (DEmRNAs) related to COAD from the TCGA database they found that ULBP2 was highly expressed in COAD tumor tissues, and the low level of ULBP2 predicted a better prognosis in COAD OS. Similarly, Demirkol et al. [52] found that ULBP2 combined with the *SEMA5A* gene could be used as a prognostic and therapeutic indicator of CC from the dataset downloaded from the GEO database. Li et al. [53] explored and analyzed the prognostic indicators of CC from the GEO database and TCGA database and found that the upregulation of the *ULBP2* gene was related to the shorter OS of CC patients. Rothe et al. [54] designed a fusion protein composed of human ULBP2 and a single chain derived from an antibody targeting tumor carcinoembryonic antigen (CEA) that could redirect NK cells to malignant cells by binding to tumor cells and NK cells, and triggered NK cell-mediated target cell killing *in vitro*.

However, our present study was certainly not without shortcomings and deficiencies. First, the clinical parameters we downloaded from the public website were not perfect (such as CA-199). Additionally, the sample size of our validation cohort was not large enough, and the number of cell validations was not large enough, which might affect our research results. Therefore, in the future, further *in vivo* and *in vitro* experiments should be performed for related investigations.

In conclusion, in this study, comprehensive analyses were conducted to determine the potential value of *NKG2DL* family genes in COAD. The mRNA expression levels of *ULBP1*, *ULBP2*, *ULBP3,* and *RAET1L* might be potential diagnostic biomarkers in COAD. The comprehensive univariate and multivariate analyses suggested that ULBP2 had prognostic value in COAD OS and RFS. We verified the prognostic value of ULBP2 from the CC-related GSE40967 dataset of the GEO database, and the results also suggested that ULBP2 had a prognostic value for the OS and RFS of CC patients. Therefore, ULBP2 might be an independent diagnostic and prognostic molecular biomarker of COAD. Finally, the verification study of the ULBP2 expression found that ULBP2 expression was relatively high in COAD tissues. We investigated the diagnostic and prognostic values of ULBP2 in COAD and found that ULBP2 had a certain diagnostic and prognostic value in COAD. Nonetheless, these still need prospective validation.

## MATERIALS AND METHODS

### Public database information mining

The expression information for the *NKG2DL* family genes and clinical parameters corresponding to COAD patients was downloaded from The Cancer Genome Atlas (TCGA) database (https://tcga-data.nci.nih.gov/, obtained on December 10, 2019) [55, 56]. Additionally, to further complete the patient's clinical parameters, we also obtained consistent clinical parameters from the database of University of California, Santa Cruz Xena browser (UCSC Xena: http://xena.ucsc.edu/, obtained on December 10, 2019) [57, 58], which shares data with the TCGA database. Finally, to validate the prognostic value of the NKG2DL genes, we downloaded the GSE40967 dataset of *NKG2DL* gene expression and corresponding clinical parameters of colon cancer (CC)-related information from the Gene Expression Omnibus database (GEO, https://www.ncbi.nlm.nih.gov/geo/query/acc.cgi?acc=GSE40967, obtained on December 10, 2019) [59].

### Bioinformatics analysis

To analyze the bio-enrichment function and metabolic pathways of the *NKG2DL* family genes, we downloaded the gene ontology (GO) term analysis and the Kyoto Encyclopedia of Genes and Genomes (KEGG) pathway analysis from the Database for Annotation, Visualization, and Integrated Discovery (DAVID) (https://david.ncifcrf.gov/; version 6.8; obtained on December 16, 2019) [60, 61]. We also investigated the GO enrichment function through the Biological Networks Gene Ontology (BiNGO) by means of Cytoscape_version 3.6.1 [62] and obtained related pathway information by downloading the database from KEGG (http://www.kegg.jp/, obtained on December 16, 2019) [63, 64]. Pearson’s correlation analyses of the *NKG2DL* genes were implemented using the co-expression matrix via the R platform. Finally, the potential correlation of *NKG2DL*s at the gene and protein levels were investigated using the public network tools of Gene Multiple Association Network Integration Algorithm (GeneMANIA, http://www.genemania.org/, obtained on December 19, 2019) [65] and Search Tool for the Retrieval of Interacting Genes (STRING, https://string-db.org/, obtained on December 19, 2019) [66, 67].

### Expression levels of *NKG2DL* genes in the normal colon tissues and COAD tumor tissues

To obtain the expression levels of *NKG2DL* genes in normal colon tissues and COAD tumor tissues, we acquired the related expression information from Genotype-Tissue Expression (GTEx) portal (https://gtexportal.org/home, obtained on December 19, 2019) [68] and Gene Expression Profiling Interactive Analysis (GEPIA; http://gepia.cancer-pku.cn/index.html; obtained on December 19, 2019) [69] respectively.

### Differential gene expression and diagnostic ROC curve analysis based on the TCGA cohort

The DESeq and edgeR [70, 71] datasets, downloaded from the TCGA database using the R platform, were used to investigate the differential expression of *NKG2DLs* in COAD through the expression of mRNAs between COAD tumor tissues and adjacent normal tissues. We considered the false discovery rate (FDR) to be < 0.05 and | log2 fold change (FC)| ≥ 2.0 was regarded as a significantly differential expression. Then, we analyzed their differential expressions and generated diagnostic ROC curves to compare COAD tumor tissues and adjacent normal tissues.

### Survival analysis

We analyzed the prognostic values of *NKG2DL* genes of COAD patients based on the TCGA cohort and also validated the prognostic values of *NKG2DL* genes in CC patients from the GSE40967 cohort. The clinical information of COAD and CC patients was integrated with the expression levels of *NKG2DL* family genes and we established univariate and multivariate survival models to investigate the prognostic values of *NKG2DL* genes in COAD and CC according to the grouping of the median expression.

### Comprehensive prognosis analysis of *NKG2DL* genes

The nomogram was performed using the R platform to analyze the contribution of prognostic clinical parameters (including prognostic genes) in COAD and CC. The greater the contribution, the higher the score, and the worse the prognosis. Besides, we investigated the correlation of prognostic genes to the TNM stage by downloading the data from the website of GEPIA. Finally, we established a prognostic risk model based on the prognostic risk score, which was the result of multiplying the expression level of the prognostic genes by the contribution coefficient (β). The relevant formula was as follows: risk score = expression of gene _1_ × β of gene _1_ + expression of gene _2_ × β _2_ of gene _2_ + …+ expression of gene _n_ × β _n_ of gene _n._ [72, 73] Based on the grouping of the median expression, a univariate survival analysis was conducted to evaluate the relevant survival in COAD and CC, while the time-dependent survival ROC was performed using the survivalROC package in the R platform [74].

### Verification of ULBP2 expression levels in COAD tumor tissue and adjacent normal tissue samples

### Patient tissue samples

The COAD tissue samples and tissue wax blocks used in this study were all obtained by surgical resection at the Colorectal and Anal Surgery Department of the First Affiliated Hospital of Guangxi Medical University. The inclusion criteria included: (1) no age and sex restrictions, (2) resection of colon adenocarcinoma tumor, and (3) pathological diagnosis of COAD. The exclusion criteria included: (1) complicated by other known tumors, (2) receiving preoperative radiotherapy and chemotherapy, (3) refusing to provide written informed consent, and iv) tumor tissue too small to obtain specimens. All subjects gave their informed consent for inclusion before they participated in the study. The study was conducted in accordance with the Declaration of Helsinki, and the protocol was approved by the Ethics Committee of the First Affiliated Hospital of Guangxi Medical University. [Ethics no.: 2020(K-Y-E-050)].

### Reverse transcription–quantitative polymerase chain reaction (RT-qPCR) of ULBP2 expressions of COAD in Guangxi cohort

After surgical removal, the tissue was quickly stored in a -80° C refrigerator. The following PCR primers were utilized: *ULBP2*, forward 5'- GAAAAGTGGGAGAATGACAAGG-3' and reverse 5'-GTCCTCAAGCCATCCTATACAG-3'; *GAPDH*, forward 5'-GTCAGCCGCATCTTCTTT-3' and reverse 5'-CGCCCAATACGACCAAAT-3'. *ULBP2* gene expression was normalized to *GAPDH* expression. TRIzol^®^ reagent was used (No. 15596026, Invitrogen) to extract total RNA from cells, followed by reverse transcription using a PrimeScript^TM^ RT reagent Kit with gDNA Eraser (Perfect Real Time, Takara Biomedical Technology (Beijing) Co., Ltd.) to obtain cDNA. Finally, we performed qPCR according to the guide of the Applied Biosystems Quantsudio^TM^ Real-Time PCR System (Q6) (Applied Biosystems, Thermo Fisher Scientific, USA). The PCR program was composed of 1 cycle at 95° C for 10 min; 40 cycles at 95° C for 15 s, 60° C for 1 min and 95° C for 30 s; 1 cycle at 95° C for 15 s, 60° C for 1 min, 95° C for 30 sec, 60° C for 15 s. The relative expression of *ULBP2* was determined using the method of 2 ^- ∆∆ Cq^ [75–77].

### Immunohistochemistry (IHC) of ULBP2 expression in COAD from the Guangxi cohort

The tissues were fixed in formalin, dehydrated, and embedded in paraffin to make wax blocks, and then sliced to a thickness of 3-4μm. To perform IHC, the tissues were deparaffinized and dehydrated. EDTA antigen retrieval was performed under high pressure for 2.5 minutes, 3% H_2_O_2_ was used to block the endogenous reaction for 5 min. Next, the ULBP2 primary antibody (No.12143-RP02, Sino Biological, Beijing, China) was incubated at 4° C overnight, and the HRP secondary antibody (No. KIT-5020, MXB Biotechnologies, Fuzhou, China) was incubated for 1 h at 25° C. Finally, a DAB kit (No. DAB-0031, MXB Biotechnologies, Fuzhou, China) was incubated for 5 min, followed by counter-staining with hematoxylin for 5 min. The scoring criteria for IHC were as follows: percentage of positive cells, 0 (0%), 1 (1–25%), 2 (26–50%), 3 (51–75%), and 4 (76– 100%); staining intensity, 0 (negative), 1 (weak), 2 (moderate) and 3 (strong). The product of the two scores was the immunohistochemical staining score [78, 79].

### Statistical analysis

All measurement data are expressed as mean ± standard deviation (x ± s). The analysis of the paired design used the paired t-test, the analysis of two random samples used the unpaired *t*-test, the comparison of means between multiple groups used single-factor variance (one-way ANOVA) analysis and the pairwise comparison of multiple samples means between samples used the SNK-q test. The log-rank test was done by the univariate Kaplan-Meier survival analysis, and univariate and multivariate survival analyses were examined on the level of the 95% confidence intervals (CIs) and hazard ratios (HRs). *P* < 0.05 was considered to be statistically different. Most of the statistical analyses were conducted using SPSS 20.0. Pearson correlation analysis, nomogram and survivalROC package were obtained using the R platform.

## Supplementary Material

Supplementary Figures

Supplementary Table 1

Supplementary Table 2

Supplementary Table 3

Supplementary Table 4

Supplementary Table 5
