# Supplementary material for: Investigation and verification of the clinical significance and perspective of natural killer group 2 member D ligands in colon adenocarcinoma
Source: Aging (Albany NY). 2021 Apr 27;13(9):12565–86. doi: 10.18632/aging.202935 (PMC8148460; doi:10.18632/aging.202935)
Supplement: Supplementary Figures [file aging-13-202935-s001.pdf]

## SUPPLEMENTARY FIGURES

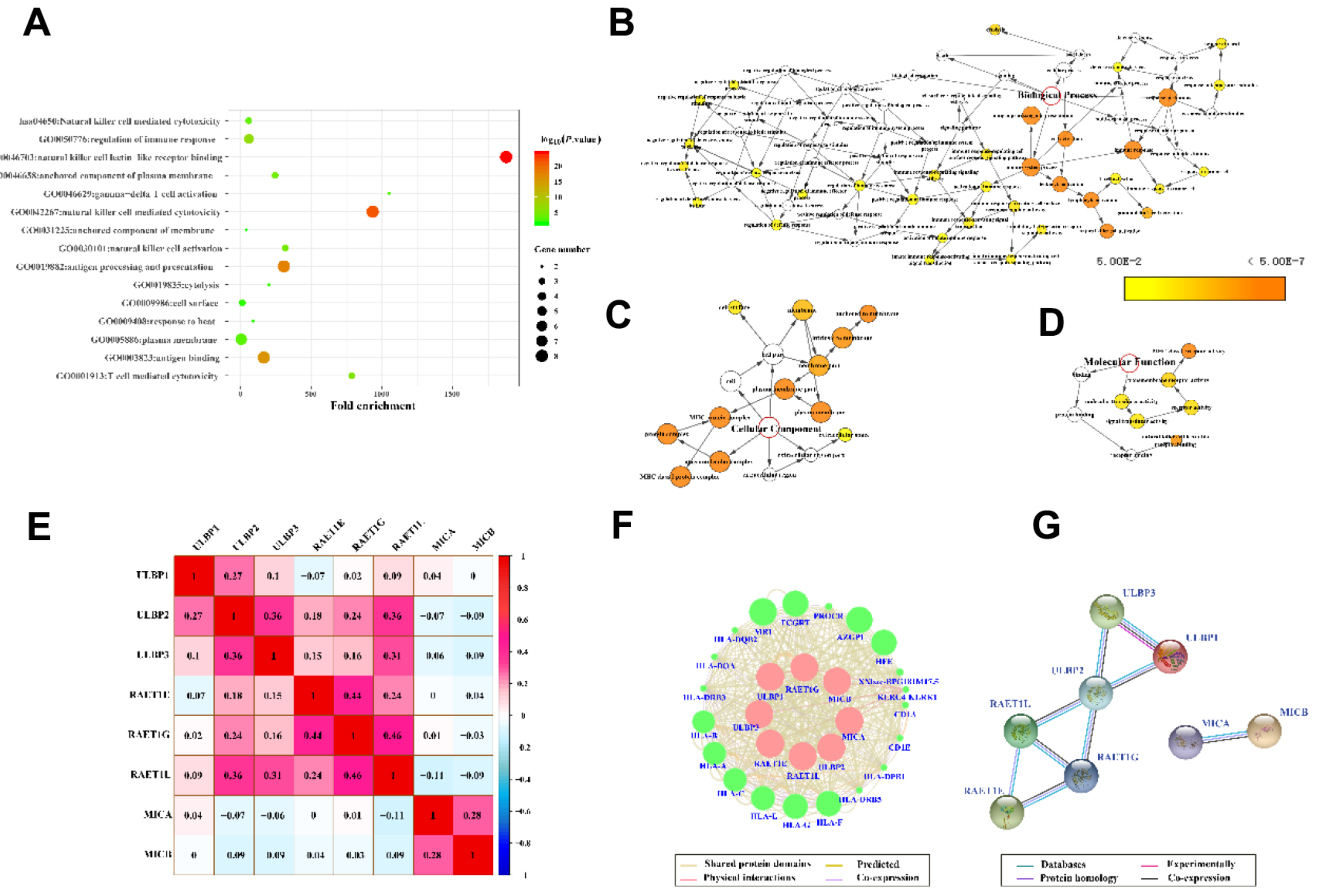


**Supplementary Figure 1. Bioinformatic analysis of *NKG2DL* family genes**. (**A**–**D**) GO term biological function analysis, KEGG analysis and Enriched Gene Ontology directed acyclic graph of *NKG2DL* family genes: (**A**) GO term biological function analysis and KEGG analysis; Gene Ontology directed acyclic graph for (**B**) Biological Process (BP), (**C**) Molecular Function (MF) and (**D**) Cellular components (CC); (**E**–**G**) Pearson's correlations analysis and gene-gene and protein-protein interaction networks of *NKG2DL* genes: (**E**) Pearson correlation analysis of *NKG2DL* genes. Red indicates a positive correlation and blue indicates a negative correlation. (**F**) GeneMANIA interaction networks; (**G**) Protein-protein interaction networks. Notes: GO, gene ontology; KEGG, Kyoto Encyclopedia of Genes and Genomes; *NKG2DL*, Natural Killer Group 2 Member D Ligand; *ULBP:* unique long 16 (UL16)-binding protein; *MIC*: Human Major Histocompatibility Complex (MHC) class I polypeptide-related sequence.

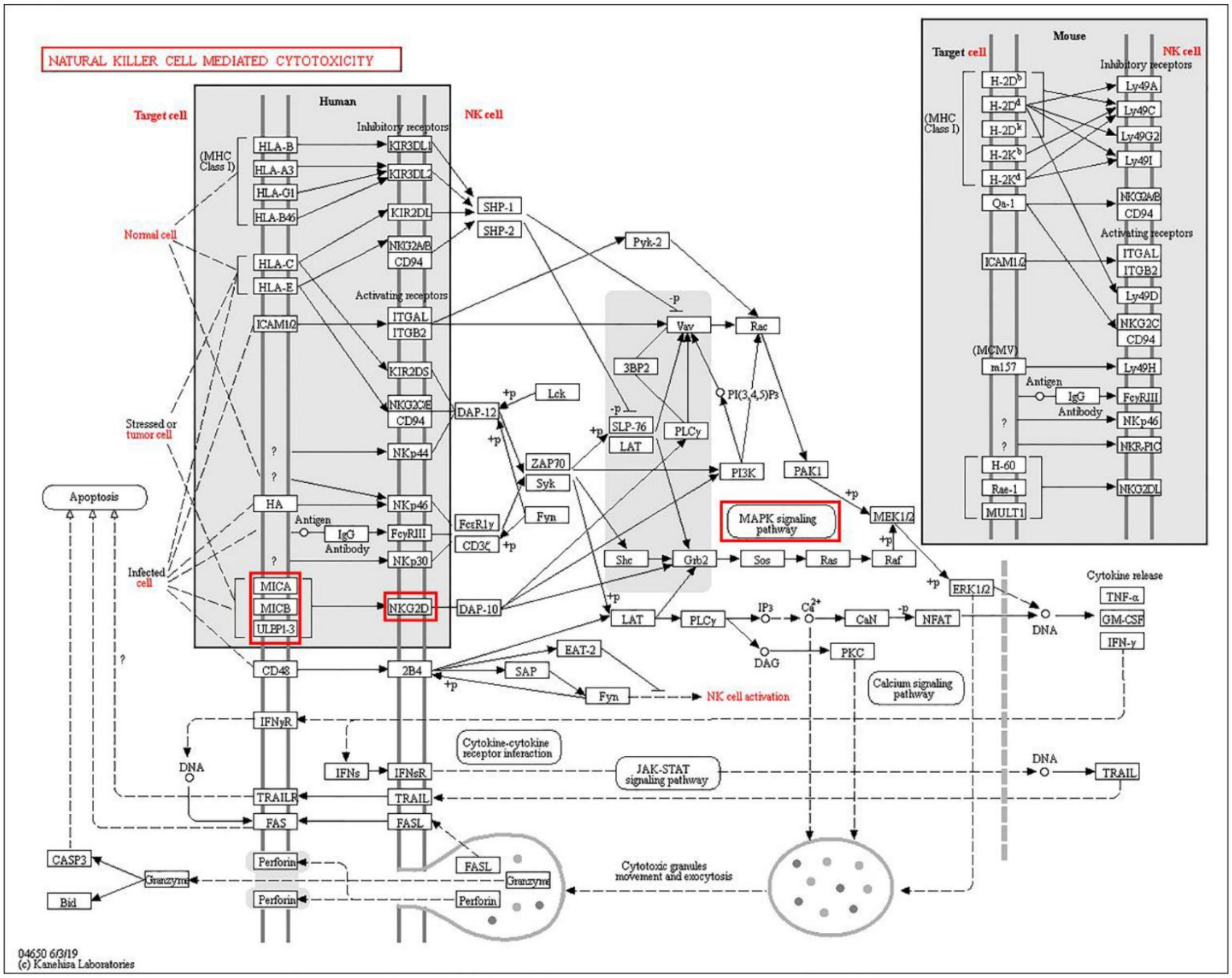


**Supplementary Figure 2. The KEGG pathway of Natural killer cell mediated cytotoxicity**. Notes: KEGG, Kyoto Encyclopedia of Genes and Genomes.

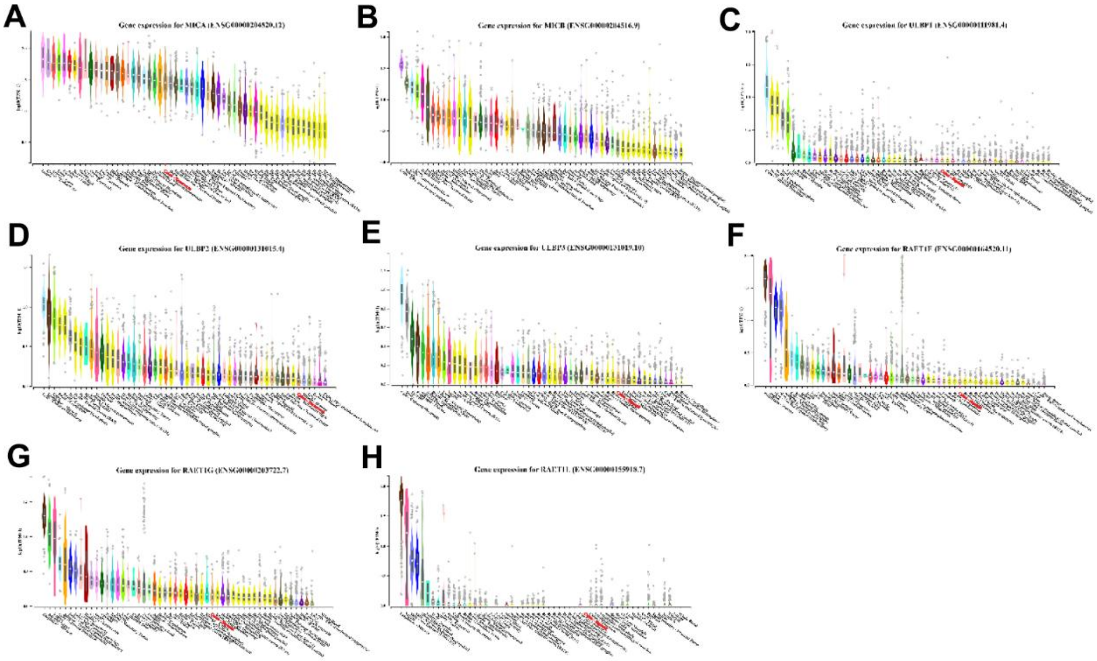


**Supplementary Figure 3. NKG2DL genes expression in normal human tissues in the GTEx database.** (**A**) *MICA*; (**B**) *MICB*; (**C**) *ULBP1*; (**D**) *ULBP2*; (**E**) *ULBP3*; (**F**) *RAET1E*; (**G**) *RAET1G*; (**H**) *RAET1L*. Notes: NKG2DL, Natural Killer Group 2 Member D Ligand.

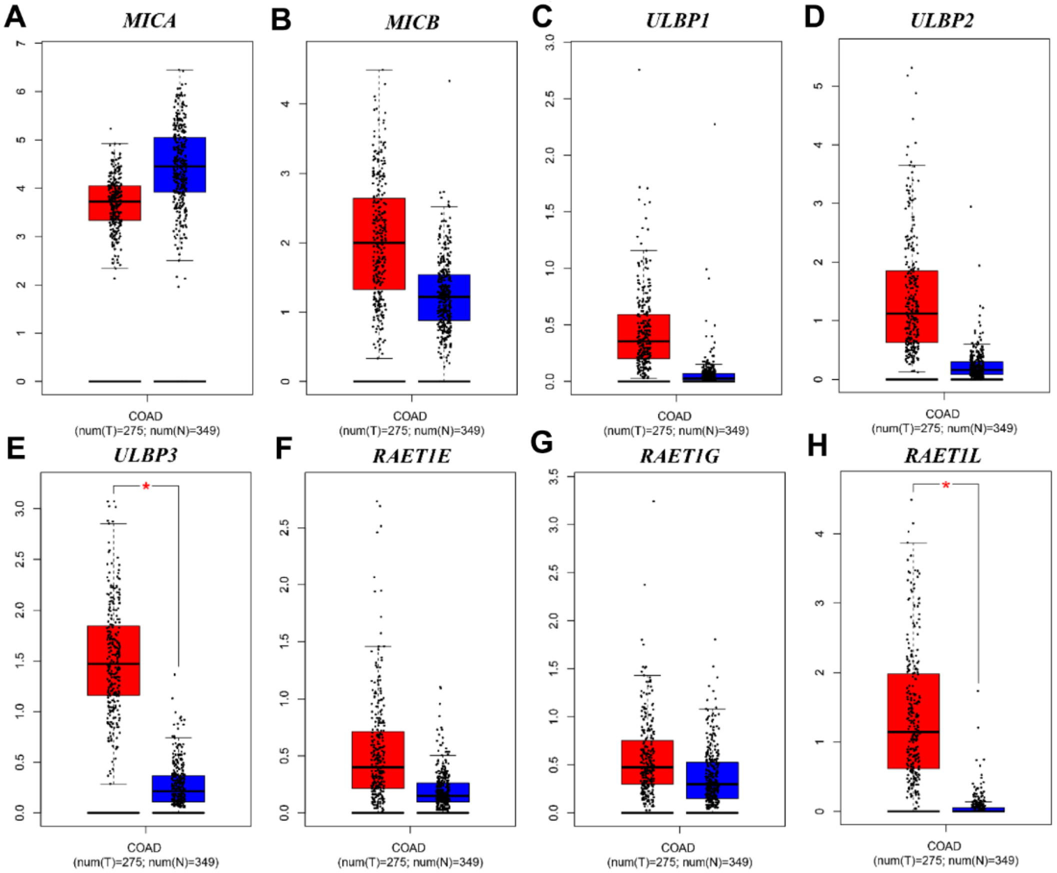


**Supplementary Figure 4. The expression level of NKG2DL genes downloaded from GEPIA website in COAD tumor tissue and normal colon tissue.** (**A**) *MICA*; (**B**) *MICB*; (**C**) *ULBP1*; (**D**) *ULBP2*; (**E**) *ULBP3*; (**F**) *RAET1E*; (**G**) *RAET1G*; (**H**) *RAET1L*. Notes: NKG2DL, Natural Killer Group 2 Member D Ligand; GEPIA, Gene Expression Profiling Interactive Analysis; COAD, colon adenocarcinoma.

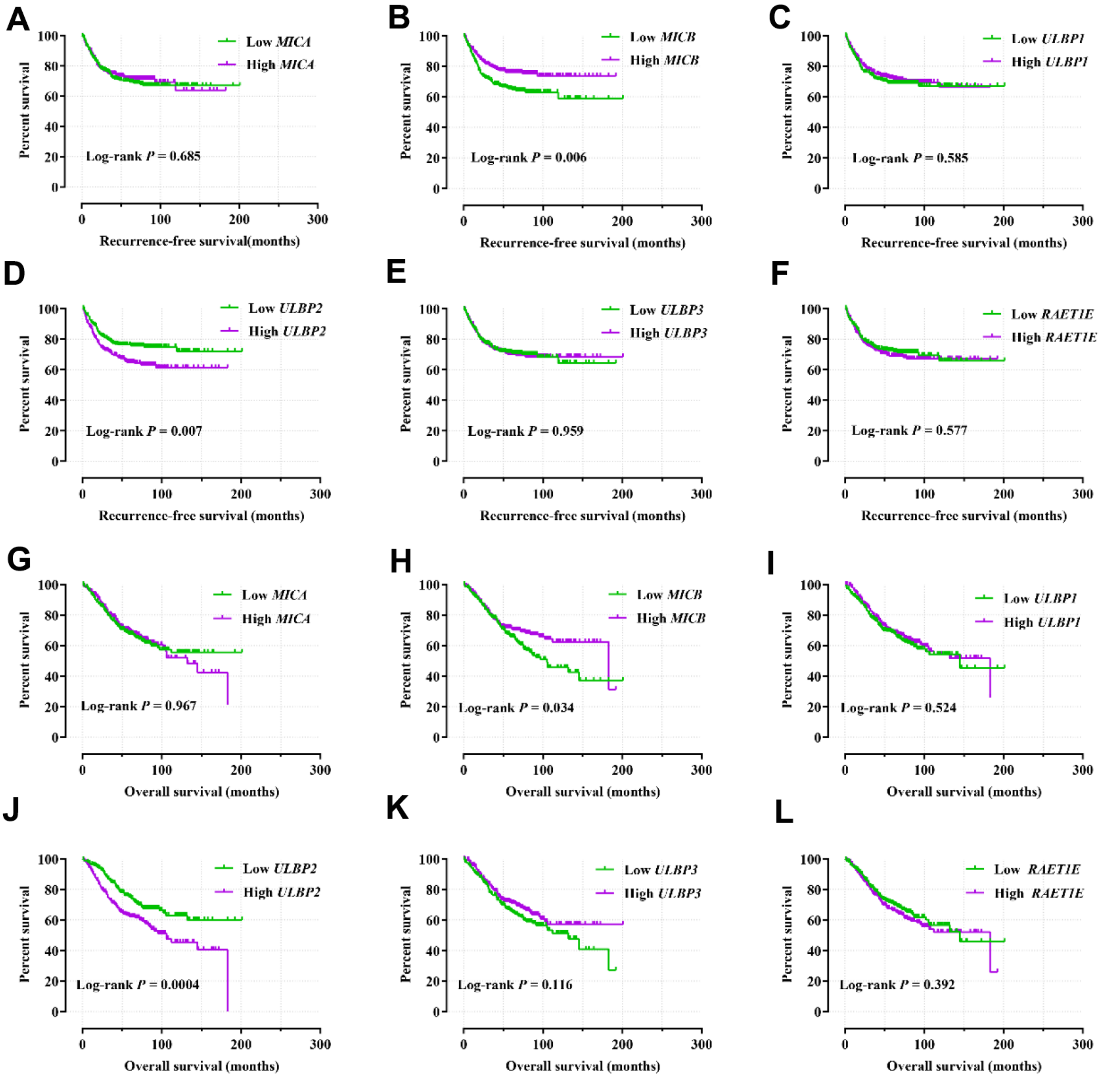


**Supplementary Figure 5. Kaplan–Meier survival curves for *NKG2DL* genes in CC of GSE40967 cohort**. Notes: RFS stratified by (**A**) *MICA*; (**B**) *MICB*; (**C**) *ULBP1*; (**D**) *ULBP2*; (**E**) *ULBP3*; (**F**) *RAET1E*. OS stratified by (**G**) *MICA*; (**H**) *MICB*; (**I**) *ULBP1*; (**J**) ULBP2; (**K**) *ULBP3*; (**L**) *RAET1E*. CC: colon cancer; *NKG2DL*, Natural Killer Group 2 Member D Ligand; *ULBP:* unique long 16 (UL16)-binding protein; *MIC*: Human Major Histocompatibility Complex (MHC) class I polypeptide-related sequence; RFS, recurrence-free survival; OS, overall survival.

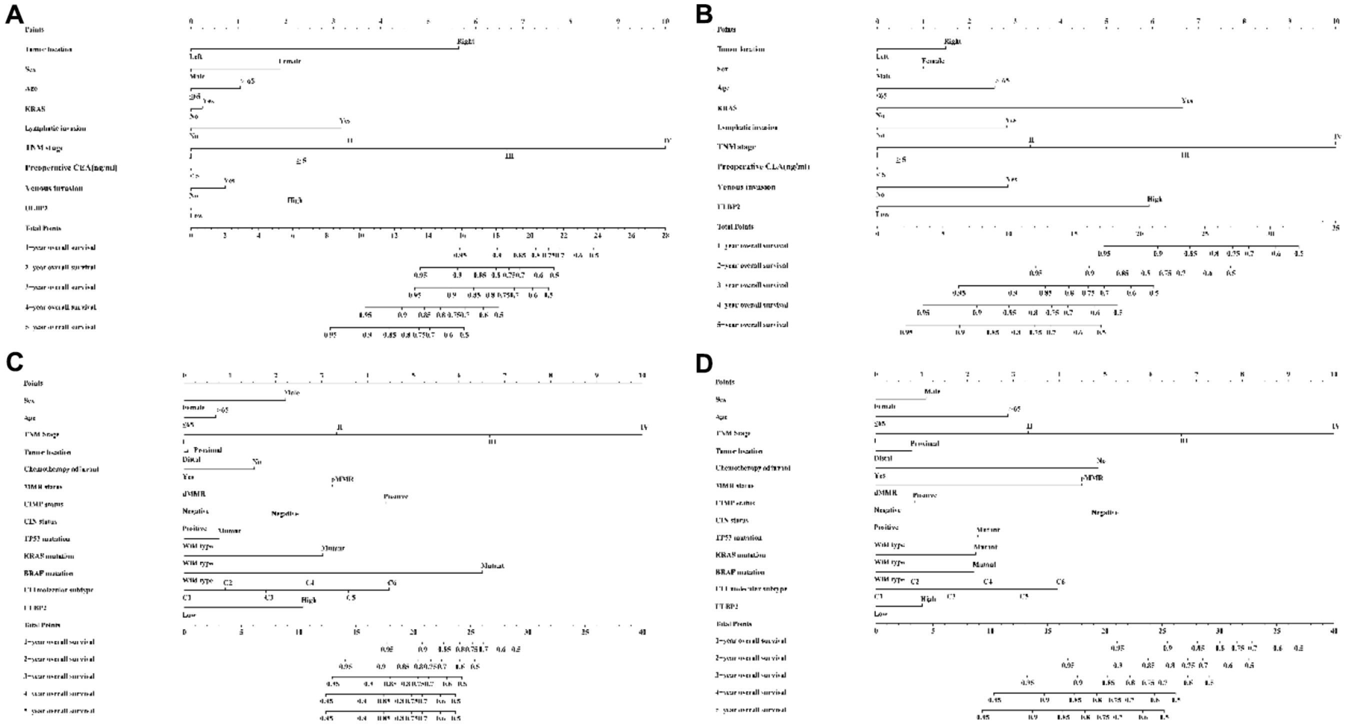


**Supplementary Figure 6. Nomogram for predicting the prognosis of COAD patients based on the TCGA cohort and GSE40967 cohort**. (**A**) The prognostic value of COAD RFS; (**B**) The prognostic value of COAD OS; (**C**) The prognostic value of CC RFS; (**D**) The prognostic value of CC OS. Notes: COAD, colon adenocarcinoma; CC: colon cancer; CEA, carcinoembryonic antigen; KRAS, Kirsten rat sarcoma viral oncogene. TNM, Tumor Node Metastasis; MMR, mismatch repair; dMMR, deficient mismatch repair, pMMR, proficient mismatch repair; CIN, chromosomal instability; TP53, tumor protein p53; BRAF, B-Raf proto-oncogene, serine/threonine kinase; CIT, Cartes d'Identité des Tumeurs; CIMP, CpG island methylator phenotype; *ULBP:* unique long 16 (UL16)-binding protein; RFS, Recurrence-free survival; OS, overall survival.

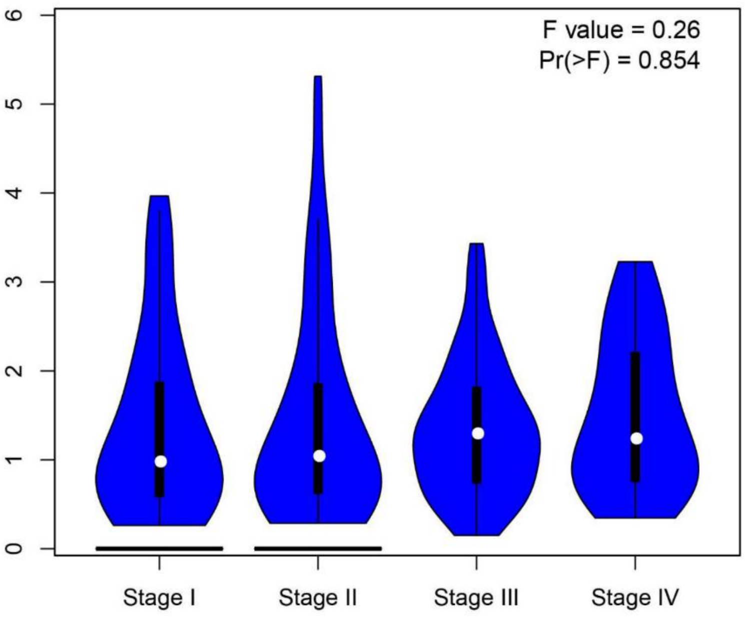


**Supplementary Figure 7. Relationship between prognosis-related ULBP2 gene and TNM stage**. Notes: COAD, colon adenocarcinoma; TNM, Tumor Node Metastasis.
