# Supplementary material for: Investigation and verification of the clinical significance and perspective of natural killer group 2 member D ligands in colon adenocarcinoma
Source: Aging (Albany NY). 2021 Apr 27;13(9):12565–86. doi: 10.18632/aging.202935 (PMC8148460; doi:10.18632/aging.202935)
Supplement: Supplementary Table 1 [file aging-13-202935-s002.doc]

Supplementary Table 1. Baseline characteristics of patients in the TCGA cohort.

|  | RFS | | | | |  | OS | | | | | | | | | | | | | | | |
| --- | --- | --- | --- | --- | --- | --- | --- | --- | --- | --- | --- | --- | --- | --- | --- | --- | --- | --- | --- | --- | --- | --- |
| Variables | Patients  (n=371) | No. of events | MST  (days) | HR (95%CI) | Log-rank *P* |  | Patients  (n=438) | No. of events | | MST  (days) | | | | HR (95%CI) | | | | | | | | Log-rank *P* |
| Age(years) |  |  |  |  |  |  |  |  | |  | | | |  | | | | | | | |  |
| ≤65 | 154 | 31 | 2564 | 1 | 0.626 |  | 175 | 43 | | 1910 | | | | 1 | | | | | | | | 0.169 |
| >65 | 215 | 46 | NA | 1.121(0.709-1.771) |  |  | 261 | 55 | | NA | | | | 1.324(0.886-1.980) | | | | | | | |  |
| Missing* | 2 |  |  |  |  |  | 2 |  | |  | | | |  | | | | | | | |  |
| Sex |  |  |  |  |  |  |  |  | |  | | | |  | | | | | | | |  |
| Male | 201 | 48 | 2270 | 1 | 0.046 |  | 234 | 54 | | 2475 | | | | 1 | | | | | | | | 0.545 |
| Female | 170 | 30 | NA | 0.629(0.398-0.996) |  |  | 204 | 44 | | NA | | | | 0.884(0.593-1.318) | | | | | | | |  |
| TNM Stage |  |  |  |  |  |  |  |  | |  | | | |  | | | | | | | |  |
| I | 61 | 5 | NA | 1 | <0.001 |  | 73 | 4 | | NA | | | | 1 | | | | | | | | <0.001 |
| II | 144 | 28 | NA | 1.934(0.745-5.020) |  |  | 168 | 28 | | 2821 | | | | 2.308(0.807-6.602) | | | | | | | |  |
| III | 109 | 25 | NA | 2.829(1.082-7.394) |  |  | 126 | 31 | | NA | | | | 4.101(1.446-11.634) | | | | | | | |  |
| IV | 47 | 18 | 1069 | 6.614(2.452-17.840) |  |  | 61 | 31 | | 858 | | | | 11.355(4.003-32.208) | | | | | | | |  |
| Missing^#^ | 10 |  |  |  |  |  | 10 |  | |  | | | |  | | | | | | | |  |
| CEA |  |  |  |  |  |  |  |  | |  | | | |  | | | | | | | |  |
| ≥5ng/mL | 81 | 24 | 1678 | 1 | 0.005 |  | 98 | 30 | | 1711 | | | | 1 | | | | | | | | <0.001 |
| < 5ng/mL | 154 | 23 | NA | 0.452(0.255-0.802) |  |  | 180 | 21 | | 3042 | | | | 0.326(0.185-0.573) | | | | | | | |  |
| Missing^&^ | 136 |  |  |  |  |  | 160 |  | |  | | | |  | | | | | | | |  |
| Tumor |  |  |  |  |  |  |  |  | |  | | | |  | | | | | | | |  |
| location |  |  |  |  |  |  |  |  | |  | | | |  | | | | | | | |  |
| Right | 212 | 41 | NA | 1 | 0.820 |  | 251 | 62 | | 2532 | | | | 1 | | | | | | | | 0.069 |
| Left | 143 | 32 | 2270 | 1.055(0.664-1.676) |  |  | 171 | 30 | | 3042 | | | | 0.669(0.433-1.035) | | | | | | | |  |
| Missing^!^ | 16 |  |  |  |  |  | 16 |  | |  | | | |  | | | | | | | |  |
| KRAS |  |  |  |  |  |  |  |  | |  | | | |  | | | | | | | |  |
| Yes | 44 | 19 | 1069 | 1 | 0.005 |  | 48 | 25 | | 1518 | | | | 1 | | | | | | | | 0.001 |
| No | 293 | 53 | 2564 | 0.474(0.277-0.809) |  |  | 355 | 58 | | 2821 | | | | 0.455(0.280-0.737) | | | | | | | |  |
| Missing% | 34 |  |  |  |  |  | 35 |  | |  | | | |  | | | | | | | |  |
| Lymphatic invasion |  |  |  |  |  |  |  |  | | | |  |  | | | | | | |  | | |
| Yes | 212 | 34 | NA | 1 | <0.001 |  | 243 | 42 | | NA | | | | | | | 1 | | | <0.001 | | |
| No | 128 | 38 | 1801 | 2.322(1.452-3.713) |  |  | 152 | 44 | | | 2003 | | | | 2.139(1.392-3.286) | | | | | |  | |
| Missing@ | 31 |  |  |  |  |  | 43 |  | | |  | | | | | |  |  | | | | |
| Venous invasion |  |  |  |  |  |  |  |  | | |  | | | | | |  |  | | | | |
| Yes | 77 | 24 | 1801 | 1 | <0.001 |  | 90 | 34 | | | 2047 | | | | | | 1 | | <0.001 | | | |
| No | 250 | 39 | NA | 0.396(0.237-0.662) |  |  | 290 | 51 | NA | | | | | | | 3.393(0.254-0.608) | | | | |  | |
| Missing | 44 |  |  |  |  |  | 58 |  | | |  | | | | | |  | | | | |  |

Notes: RFS: recurrence-free survival; OS: overall survival; Missing*, information of age were unknown both RFS and OS in 2 patients; Missing^#^: information of TNM stage was unknown both RFS and OS in 10 patients; Missing^&^: information of CEA were unavailable in 136 patients of RFS and 160 patients of OS; Missing^!^: information of tumor location was unknown both RFS and OS in 16 patients; Missing%: information of KRAS were unavailable in 34 patients of RFS and 35 patients of OS; Missing@: information of Lymphatic invasion were not reported in 31 patients of RFS and 43 patients of OS. Missing: information of Venous invasion was not reported in 44 patients of RFS and 58 patients of OS TCGA: The Cancer Genome Atlas; MST: median survival time; 95 % CI: 95 % confidence interval; HR: hazards ratio; NA: not available; TNM: Tumor Node Metastasis; CEA: carcino-embryonic antigen; KRAS: Kirsten rat sarcoma viral oncogene.
