# Supplementary material for: Investigation and verification of the clinical significance and perspective of natural killer group 2 member D ligands in colon adenocarcinoma
Source: Aging (Albany NY). 2021 Apr 27;13(9):12565–86. doi: 10.18632/aging.202935 (PMC8148460; doi:10.18632/aging.202935)
Supplement: Supplementary Table 2 [file aging-13-202935-s003.doc]

Supplementary Table 2. Baseline characteristics of patients in the GSE40967 cohort.

|  | RFS | | | |  | OS | | | | | | | | | | | | | | | | | | | | | | | | | |  |
| --- | --- | --- | --- | --- | --- | --- | --- | --- | --- | --- | --- | --- | --- | --- | --- | --- | --- | --- | --- | --- | --- | --- | --- | --- | --- | --- | --- | --- | --- | --- | --- | --- |
| Variables | Patients  (n=574) | No. of events | MST  (months) | HR (95%CI) | | | Log-rank *P* | | |  | Patients  (n=579) | | | | | | No. of events | | | | | MST  (months) | | | | HR (95%CI) | | | Log-rank *P* | | |  |
| Age(years) |  |  |  |  | | |  | | |  |  | | | | | |  | | | | |  | | | |  | | |  | | |  |
| ≤65 | 226 | 78 | NA | 1 | | | 0.255 | | |  | 227 | | | | | | 63 | | | | | NA | | | | 1 | | | 0.010 | | |  |
| >65 | 347 | 101 | NA | 0.844(0.628-1.134) | | |  | | |  | 351 | | | | | | 131 | | | | | 105 | | | | 1.479(1.094-1.999) | | |  | | |  |
| Missing* | 1 |  |  |  | | |  | | |  | 1 | | | | | |  | | | | |  | | | |  | | |  | | |  |
| Sex |  |  |  |  | | |  | | |  |  | | | | | |  | | | | |  | | | |  | | |  | | |  |
| Male | 317 | 108 | NA | 1 | | | 0.096 | | |  | 319 | | | | | | 117 | | | | | 112 | | | | 1 | | | 0.066 | | |  |
| Female | 257 | 71 | NA | 0.778(0.576-1.049) | | |  | | |  | 260 | | | | | | 77 | | | | | 183 | | | | 0.765(0.573-1.020) | | |  | | |  |
| TNM Stage |  |  |  |  | | |  | | |  |  | | | | | |  | | | | |  | | | |  | | |  | | |  |
| I | 41 | 1 | NA | 1 | | | <0.001 | | |  | 41 | | | | | | 6 | | | | | NA | | | | 1 | | | <0.001 | | |  |
| II | 267 | 61 | NA | 9.896(1.372-71.383) | | |  | | |  | 269 | | | | | | 80 | | | | | 183 | | | | 1.917(0.836-4.396) | | |  | | |  |
| III | 206 | 80 | NA | 2.538(2.538-131.064) | | |  | | |  | 209 | | | | | | 68 | | | | | NA | | | | 2.319(1.006-5.346) | | |  | | |  |
| IV | 60 | 37 | 1 | 67.285(9.209-491.616) | | |  | | |  | 60 | | | | | | 40 | | | | | 27 | | | | 9.576(4.045-22.672) | | |  | | |  |
| Chemotherapy adjuvant |  |  |  |  | | |  | | |  |  | | | | | |  | | | | |  | | | |  | | |  | | |  |
| No | 319 | 73 | NA | 1 | | | <0.001 | | |  | 323 | | | | | | 107 | | | | | 183 | | | | 1 | | | 0.607 | | |  |
| Yes | 239 | 91 | NA | 1.752(1.287-2.384) | | |  | | |  | 240 | | | | | | 76 | | | | | 145 | | | | 0.926(0.690-1.243) | | |  | | |  |
| Missing^&^ | 16 |  |  |  | | |  | | |  | 16 | | | | | |  | | | | |  | | | |  | | |  | | |  |
| Tumor |  |  |  |  | | |  | | |  |  | | | | | |  | | | | |  | | | |  | | |  | | |  |
| location |  |  |  |  | | |  | | |  |  | | | | | |  | | | | |  | | | |  | | |  | | |  |
| Distal | 348 | 118 | NA | 1 | | | 0.107 | | |  | 351 | | | | | | 118 | | | | | 145 | | | | 1 | | | 0.584 | | |  |
| Proximal | 226 | 61 | NA | 0.777(0.571-1.059) | | |  | | |  | 228 | | | | | | 76 | | | | | NA | | | | 1.084(0.812-1.447) | | |  | | |  |
| KRAS mutation |  |  |  |  | | |  | | |  |  | | | | | |  | | | | |  | | | |  | | |  | | |  |
| Mutant | 214 | 78 | NA | 1 | | | 0.005 | | |  | 216 | | | | | | 81 | | | | | 132 | | | | 1 | | | 0.037 | | |  |
| Wild type | 322 | 91 | NA | 0.707(0.523-0.958) | | |  | | |  | 325 | | | | | | 106 | | | | | 145 | | | | 0.736(0.551-0.983) | | |  | | |  |
| Missing% | 38 |  |  |  | | |  | | |  | 38 | | | | | |  | | | | |  | | | |  | | |  | | |  |
| MMR status |  |  |  |  | | |  | | |  |  | | |  | | | | | | |  | | | |  | | | | | | |  |
| dMMR | 74 | 11 | NA | 1 | | | 0.002 | | |  | 74 | | | | | | 19 | | | | | NA | | | | 1 | | 0.397 | | | |  |
| pMMR | 454 | 158 | NA | 2.495(1.354-4.598) | | |  | | |  | 459 | | | | | | 160 | | | | | NA | | | | 1.227(0.762-1.977) | |  | | | |  |
| Missing@ | 46 |  |  |  | | |  | | |  | 46 | | | | | |  | | | | |  | | | |  | | |  | | |  |
| CIN status |  |  |  |  | | |  | | |  |  | | | | | |  | | | | |  | | | |  | | |  | | |  |
| Negative | 107 | 24 | NA | 1 | | | 0.609 | | |  | 110 | | | | | | 36 | | | | | NA | | | | 1 | | | 0.170 | | |  |
| Positive | 365 | 99 | NA | 1.123(0.718-1.754) | | |  | | |  | 367 | | | | | | 116 | | | | | 145 | | | | 0.770(0.529-1.121) | | |  | | |  |
| Missing^#^ | 102 |  |  |  | | |  | | |  | 102 | | | | | |  | | | | |  | | | |  | | |  | | |  |
| TP53 mutation |  |  |  |  | | |  | | |  |  | | | | | |  | | | | |  | | | |  | | |  | | |  |
| Mutant | 188 | 79 | NA | 1 | | | 0.085 | | |  | 190 | | | | | | 78 | | | | | 105 | | | | 1 | | | 0.312 | | |  |
| Wild type | 156 | 51 | NA | 1.737(0.518-1.048) | | |  | | |  | 159 | | | | | | 53 | | | | | NA | | | | 0.836(0.590-1.185) | | |  | | |  |
| Missing^!^ | 230 |  |  |  | | |  | | |  | 230 | | | | | |  | | | | |  | | | |  | | |  | | |  |
| BRAF mutation |  |  |  |  | | |  | | |  | |  | | | | | |  | | | | | |  | | |  | |  | | |  |
| Mutant | 49 | 13 | NA | 1 | | | 0.808 | | |  | 49 | | | | | | 16 | | | | | NA | | | | | 1 | | 0.689 | | |  |
| Wild type | 453 | 137 | NA | 1.072(0.607-1.895) | | |  | | |  | 458 | | | | | | 156 | | | | | 145 | | | | | 0.900(0.538-1.508) | |  | | |  |
| Missing? | 72 |  |  |  | | |  | | |  | 72 | | | | | |  | | | | |  | | | | |  | |  | | |  |
| CIT molecular subtype |  |  |  |  | | |  | | |  |  | | | | | |  | | | | |  | | | | |  | |  | | |  |
| C1 | 114 | 37 | NA | 1 | | | <0.001 | | |  | 116 | | | | | | 42 | | | | | 86 | | | | | 1 | | 0.002 | | |  |
| C2 | 101 | 19 | NA | 0.524(0.302-0.912) | | |  | | |  | 101 | | | | | | 28 | | | | | NA | | | | | 0.722(0.447-1.165) | |  | | |  |
| C3 | 70 | 19 | NA | 0.817(0.470-1.420) | | |  | | |  | 73 | | | | | | 16 | | | | | NA | | | | | 0.639(0.360-1.137) | |  | | |  |
| C4 | 59 | 29 | 41 | 1.921(1.181-3.125) | | |  | | |  | 59 | | | | | | 31 | | | | | 46 | | | | | 1.790(1.125-2.850) | |  | | |  |
| C5 | 152 | 47 | NA | 0.922(0.599-1.418) | | |  | | |  | 152 | | | | | | 51 | | | | | 145 | | | | | 0.855(0.567-1.288) | |  | | |  |
| C6 | 60 | 26 | NA | 1.394(0.844-2.302) | | |  | | |  | 60 | | | | | | 23 | | | | | 105 | | | | | 1.001(0.602-1.665) | |  | | |  |
| Missing^+^ | 18 |  |  |  | | |  | | |  | 18 | | | | | |  | | | | |  | | | | |  | |  | | |  |
| CIMP status |  |  |  |  | | |  | | |  | |  | | | |  | | | |  | | | | |  | | | | | | | |
| Negative | 413 | 124 | NA | 1 | | | 0.215 |  | | 418 | | | 138 | | | | | | | | | 145 | | | | | 1 | | 0.589 | | |  |
| Positive | 91 | 20 | NA | 0.745(0.464-1.194) | | |  | | 91 | | | | 30 | | | | | | | | | NA | | | | | 1.115(0.751-1.656) | | |  |  | |
| Missing^-^ | 40 |  |  |  | | |  | | | | 40 | | | |  | | | |  | | | |  | | | | | | | | |  |

Notes: RFS, recurrence-free survival; OS, overall survival; Missing*, information of age was unknown both RFS and OS in 1 patient; Missing^&^, information of Chemotherapy adjuvant were unavailable both RFS and OS in 16 patients; Missing%, information of KRAS mutation were unavailable both RFS and OS in 38 patients; Missing@, information of MMR status were not reported both RFS and OS in 46 patients. Missing^#^, information of CIN status were unknown both RFS and OS in 102 patients; Missing^!^, information of TP53 mutation was unknown both RFS and OS in 230 patients; Missing?, information of BRAF mutation was unavailable both RFS and OS in 72 patients; Missing^+^, information of CIT molecular subtype were unavailable both RFS and OS in 18 patients; Missing^-^, information of CIMP status were unavailable both RFS and OS in 40 patients; MST, median survival time; 95 % CI, 95 % confidence interval; HR, hazards ratio; NA, not available; TNM, Tumor Node Metastasis; KRAS, Kirsten rat sarcoma viral oncogene; MMR, mismatch repair; dMMR, deficient mismatch repair, pMMR, proficient mismatch repair; CIN, chromosomal instability; TP53, tumor protein p53；BRAF, B-Raf proto-oncogene, serine/threonine kinase; CIT, Cartes d'Identité des Tumeurs; CIMP, CpG island methylator phenotype.
