# Supplementary material for: Investigation and verification of the clinical significance and perspective of natural killer group 2 member D ligands in colon adenocarcinoma
Source: Aging (Albany NY). 2021 Apr 27;13(9):12565–86. doi: 10.18632/aging.202935 (PMC8148460; doi:10.18632/aging.202935)
Supplement: Supplementary Table 3 [file aging-13-202935-s004.doc]

Supplementary Table 3. Prognostic values of *NKG2DL* family genes expression in COAD of the TCGA cohort.

| Gene expression | RFS | | | | | | |  | OS | | | | | | |
| --- | --- | --- | --- | --- | --- | --- | --- | --- | --- | --- | --- | --- | --- | --- | --- |
|  | Patients  (n=371) | No. of events | MST  (days) | Crude HR  (95% CI) | Crude  *P* | Adjusted HR  (95% CI) | Adjusted  *P* § |  | Patients  (n=438) | No. of events | MST  (days) | Crude HR  (95% CI) | Crude *P* | Adjusted HR  (95% CI) | Adjusted  *P* & |
| *MICA* |  |  |  |  |  |  |  |  |  |  |  |  |  |  |  |
| Low | 186 | 38 | 2564 | 1 | 0.685 | 1 | 0.052 |  | 219 | 46 | 2821 | 1 | 0.343 | 1 | 0.296 |
| High | 185 | 40 | NA | 1.096(0.703-1.710) |  | 0.462(0.212-1.007) |  |  | 219 | 52 | 2532 | 1.212(0.814-1.804) |  | 1.468（0.715-3.017） |  |
| *MICB* |  |  |  |  |  |  |  |  |  |  |  |  |  |  |  |
| Low | 186 | 34 | NA | 1 | 0.328 | 1 | 0.116 |  | 219 | 46 | 2821 | 1 | 0.705 | 1 | 0.886 |
| High | 185 | 44 | 2394 | 1.250(0.798-1.956) |  | 1.880(0.857-4.124) |  |  | 219 | 52 | 2532 | 1.080(0.725-1.607) |  | 1.054（0.515-2.158） |  |
| *ULBP1* |  |  |  |  |  |  |  |  |  |  |  |  |  |  |  |
| Low | 186 | 40 | 2564 | 1 | 0.693 | 1 | 0.302 |  | 219 | 43 | 3042 | 1 | 0.045 | 1 | 0.334 |
| High | 185 | 38 | NA | 1.094(0.701-1.707) |  | 0.667(0.309-1.439) |  |  | 219 | 55 | 2532 | 1.502(1.007-2.241) |  | 1.428(0.694-2.939) |  |
| *ULBP2* |  |  |  |  |  |  |  |  |  |  |  |  |  |  |  |
| Low | 186 | 32 | NA | 1 | 0.029 | 1 | 0.007 |  | 219 | 39 | 3042 | 1 | 0.004 | 1 | 0.003 |
| High | 185 | 46 | 2394 | 1.647(1.047-2.590) |  | 2.940(1.349-6.405) |  |  | 219 | 59 | 2047 | 1.799(1.198-2.702) |  | 3.199(1.479-6.922) |  |
| *ULBP3* |  |  |  |  |  |  |  |  |  |  |  |  |  |  |  |
| Low | 186 | 42 | 2394 | 1 | 0.354 | 1 | 0.584 |  | 219 | 52 | 2821 | 1 | 0.770 | 1 | 0.420 |
| High | 185 | 36 | NA | 0.811(0.519-1.265) |  | 1.235(0.580-2.631) |  |  | 219 | 46 | 2475 | 0.942(0.633-1.402) |  | 1.352(0.650-2.811) |  |
| *RAETE1E* |  |  |  |  |  |  |  |  |  |  |  |  |  |  |  |
| Low | 186 | 46 | 2270 | 1 | 0.053 | 1 | 0.009 |  | 219 | 55 | 2532 | 1 | 0.130 | 1 | 0.088 |
| High | 185 | 32 | NA | 0.639(0.407-1.004) |  | 0.236(0.157-0.764) |  |  | 219 | 43 | 2475 | 0.736(0.493-1.097) |  | 0.530(0.256-1.099) |  |
| *RAETE1G* |  |  |  |  |  |  |  |  |  |  |  |  |  |  |  |
| Low | 186 | 37 | 2564 | 1 | 0.570 | 1 | 0.787 |  | 219 | 48 | 3042 | 1 | 0.644 | 1 | 0.377 |
| High | 185 | 41 | NA | 1.137(0.729-1.775) |  | 1.107(0.530-2.309) |  |  | 219 | 50 | 2134 | 1.098(0.739-1.633) |  | 1.372(0.680-2.769) |  |
| *RAETE1L* |  |  |  |  |  |  |  |  |  |  |  |  |  |  |  |
| Low | 186 | 40 | 2270 | 1 | 0.460 | 1 | 0.550 |  | 219 | 49 | 2475 | 1 | 0.939 | 1 | 0.309 |
| High | 185 | 38 | NA | 0.846(0.542-1.320) |  | 0.803(0.392-1.647) |  |  | 219 | 49 | NA | 0.980(0.662-1.465) |  | 0.687(0.333-1.415) |  |

Notes: Adjusted P ^§^, adjustment for Gender, TNM Stage, CEA, KRAS, Lymphatic invasion, and Venous invasion; Adjusted P ^&^, adjustment for TNM Stage, CEA, KRAS， Lymphatic invasion and Venous invasion; COAD, colon adenocarcinoma; *NKG2DL, Natural Killer Group 2 Member D Ligand;* TCGA, The Cancer Genome Atlas; OS, overall survival; RFS, recurrence-free survival; MST, median survival time; HR, hazard ratio; CI, confidence interval; NA, not available; CEA, carcino-embryonic antigen; KRAS: Kirsten rat sarcoma viral oncogene; TNM, Tumor Node Metastasis.
