# Supplementary material for: Investigation and verification of the clinical significance and perspective of natural killer group 2 member D ligands in colon adenocarcinoma
Source: Aging (Albany NY). 2021 Apr 27;13(9):12565–86. doi: 10.18632/aging.202935 (PMC8148460; doi:10.18632/aging.202935)
Supplement: Supplementary Table 4 [file aging-13-202935-s005.doc]

Supplementary Table 4. Prognostic values of *NKG2DL* family genes expression in CC of GSE40967 cohort.

| Gene expression | RFS | | | | | | |  | OS | | | | | | |
| --- | --- | --- | --- | --- | --- | --- | --- | --- | --- | --- | --- | --- | --- | --- | --- |
|  | Patients  (n=574) | No. of events | MST  (days) | Crude HR  (95% CI) | Crude  *P* | Adjusted HR  (95% CI) | Adjusted  *P* § |  | Patients  (n=579) | No. of events | MST  (days) | Crude HR  (95% CI) | Crude *P* | Adjusted HR  (95% CI) | Adjusted  *P* & |
| *MICA* |  |  |  |  |  |  |  |  |  |  |  |  |  |  |  |
| Low | 287 | 93 | NA | 1 | 0.603 | 1 | 0.489 |  | 290 | 99 | NA | 1 | 0.854 | 1 | 0.164 |
| High | 287 | 86 | NA | 0.926 (0.690-1.241) |  | 0.890(0.639-1.239) |  |  | 289 | 95 | 132 | 0.974(0.735-1.291) |  | 1.248(0.914-1.706) |  |
| *MICB* |  |  |  |  |  |  |  |  |  |  |  |  |  |  |  |
| Low | 287 | 102 | NA | 1 | 0.027 | 1 | 0.318 |  | 290 | 108 | 108 | 1 | 0.050 | 1 | 0.037 |
| High | 287 | 77 | NA | 0.718(0.534-0.966) |  | 0.844(0.606-1.177) |  |  | 289 | 86 | 86 | 0.755(0.568-1.002) |  | 0.722(0.532-0.980) |  |
| *ULBP1* |  |  |  |  |  |  |  |  |  |  |  |  |  |  |  |
| Low | 287 | 93 | NA | 1 | 0.342 | 1 | 0.745 |  | 290 | 97 | 145 | 1 | 0.627 | 1 | 0.983 |
| High | 287 | 86 | NA | 0.869(0.648-1.165) |  | 0.949(0.691-1.303) |  |  | 289 | 97 | 183 | 0.933(0.704-1.236) |  | 1.003(0.749-1.343) |  |
| *ULBP2* |  |  |  |  |  |  |  |  |  |  |  |  |  |  |  |
| Low | 287 | 77 | NA | 1 | 0.015 | 1 | 0.036 |  | 290 | 79 | NA | 1 | <0.001 | 1 | 0.005 |
| High | 287 | 102 | NA | 1.439(1.070-1.935) |  | 1.423(1.024-1.979) |  |  | 289 | 115 | 106 | 1.667(1.251-2.220) |  | 1.563(1.146-2.130) |  |
| *ULBP3* |  |  |  |  |  |  |  |  |  |  |  |  |  |  |  |
| Low | 287 | 91 | NA | 1 | 0.578 | 1 | 0.398 |  | 290 | 107 | 132 | 1 | 0.089 | 1 | 0.082 |
| High | 287 | 88 | NA | 0.921(0.687-1.234) |  | 0.872(0.635-1.198) |  |  | 289 | 87 | NA | 0.783(0.590-1.040) |  | 0.769(0.573-1.033) |  |
| *RAETE1E* |  |  |  |  |  |  |  |  |  |  |  |  |  |  |  |
| Low | 287 | 89 | NA | 1 | 0.924 | 1 | 0.783 |  | 290 | 92 | 145 | 1 | 0.397 | 1 | 0.336 |
| High | 287 | 90 | NA | 0.986(0.736-1.322) |  | 1.046(0.758-1.443) |  |  | 289 | 102 | 183 | 1.129(0.852-1.497) |  | 1.155(0.861-1.548) |  |

Notes: Adjusted P ^§^, adjustment for TNM Stage, Chemotherapy adjuvant, KRAS mutation, MMR status, and CIT molecular subtype; Adjusted P ^&^, adjustment for Age, TNM Stage, KRAS mutation and CIT molecular subtype; CC, colon cancer; *MIC*, MHC class I polypeptide-related sequence; *NKG2DL, Natural Killer Group 2 Member D Ligand*; OS, overall survival; RFS, recurrence-free survival; MST, median survival time; HR, hazard ratio; CI, confidence interval; NA, not available; TNM, Tumor Node Metastasis; KRAS, Kirsten rat sarcoma viral oncogene; MMR, mismatch repair; CIT, Cartes d'Identité des Tumeurs.
