# Supplementary material for: Investigation and verification of the clinical significance and perspective of natural killer group 2 member D ligands in colon adenocarcinoma
Source: Aging (Albany NY). 2021 Apr 27;13(9):12565–86. doi: 10.18632/aging.202935 (PMC8148460; doi:10.18632/aging.202935)
Supplement: Supplementary Table 5 [file aging-13-202935-s006.doc]

Supplementary Table 5. Baseline parameters of COAD patients and the prognostic value of *ULBP2* in COAD.

| Variable | |  |  |  | OS* |  |  |  |  |  | RFS^#^ |  |
| --- | --- | --- | --- | --- | --- | --- | --- | --- | --- | --- | --- | --- |
|  |  | Patients  (n=159) | No. of events | MST (days) | HR (95% CI) | Log-rank *P* |  | Patients  (n=132) | No. of events | MRT (days) | HR (95% CI) | Log-rank *P* |
| Sex |  |  |  |  |  | 0.348 |  |  |  |  |  | 0.358 |
|  | Male | 97 | 27 | NA | 1 |  |  | 79 | 12 | NA | 1 |  |
|  | Female | 62 | 13 | NA | 0.729(0.376-1.414) |  |  | 53 | 5 | NA | 0.616 (0.207-1.748) |  |
| Age (years) |  |  |  |  |  | 0.626 |  |  |  |  |  | 0.117 |
|  | ≤65 | 105 | 25 | NA | 1 |  |  | 85 | 8 | NA | 1 |  |
|  | >65 | 54 | 15 | NA | 1.173 (0.618-2.224) |  |  | 47 | 9 | NA | 2.104 (0.812-5.453) |  |
| Tumor location |  |  |  |  |  | 0.413 |  |  |  |  |  | 0.588 |
|  | Right | 80 | 18 | NA | 1 |  |  | 70 | 8 | NA | 1 |  |
|  | Left | 79 | 22 | NA | 1.296(0.695-2.418) |  |  | 62 | 9 | NA | 1.300 (0.501-3.370) |  |
| TNM stage |  |  |  |  |  | <0.0001 |  |  |  |  |  | 0.049 |
|  | I | 25 | 2 | NA | 1 |  |  | 25 | 2 | NA | 1 |  |
|  | II | 58 | 4 | NA | 0.847(0.155-4.627) |  |  | 56 | 4 | NA | 0.881 (0.161-4.812) |  |
|  | III | 53 | 12 | NA | 3.110(0.696-13.902) |  |  | 51 | 11 | NA | 2.982 (0.661-13.456) |  |
|  | IV | 23 | 22 | 481 | 46.107(10.452-203.386) |  |  | 0 |  |  |  |  |
| Tumor type ^a^ |  |  |  |  |  | 0.861 |  |  |  |  |  | 0.706 |
|  | Invasive | 7 | 2 | NA | 1 |  |  | 5 | 0 | NA | 1 |  |
|  | Ulcerative | 110 | 29 | NA | 0.879 (0.210-3.685) |  |  | 90 | 90 | NA | 8737.847[0-(1.646E+099)] |  |
|  | Mass | 36 | 8 | NA | 0.722(0.153-3.401) |  |  | 31 | 31 | NA | 8474.661[0-(1.599E+099)] |  |
|  | Missing | 6 |  |  |  |  |  | 6 |  |  |  |  |
| Tumor  differentiation |  |  |  |  |  | 0.186 |  |  |  |  |  | 0.948 |
|  | Well | 18 | 7 | NA | 1 |  |  | 13 | 2 | NA | 1 |  |
|  | Moderate | 132 | 32 | NA | 0.528(0.233-1.196) |  |  | 110 | 14 | NA | 0.797 (0.181-3.508) |  |
|  | Poor | 9 | 1 | NA | 3.076 (0.029-1.896) |  |  | 9 | 1 | NA | 0.721 (0.065-7.959) |  |
| Tumor thrombus |  |  |  |  |  | <0.0001 |  |  |  |  |  | 0.181 |
|  | No | 143 | 29 | NA | 1 |  |  | 125 |  | NA | 1 |  |
|  | Yes | 16 | 11 | 601 | 5.720(2.832-11.552) |  |  | 7 |  | NA | 2.635 (0.602-11.525) |  |
| Tumor size (cm) ^b^ |  |  |  |  |  | 0.086 |  |  |  |  |  | 0.205 |
|  | <5 | 71 | 23 | NA | 1 |  |  | 57 | 10 | NA | 1 |  |
|  | ≥5 | 84 | 17 | NA | 0.581(0.310-1.088) |  |  | 71 | 7 | NA | 0.540 (0.206-1.420) |  |
|  | Missing | 4 |  |  |  |  |  | 4 |  |  |  |  |
| Lymph invasion |  |  |  |  |  | <0.0001 |  |  |  |  |  | 0.007 |
|  | No | 64 | 27 | NA | 1 |  |  | 48 | 11 | NA | 1 |  |
|  | Yes | 95 | 13 | NA | 3.708(1.911-7.197) |  |  | 84 | 6 | NA | 3.621 (1.338-9.795) |  |
| Radical resection |  |  |  |  |  | <0.0001 |  |  |  |  |  |  |
|  | Yes | 133 | 17 | NA | 1 |  |  |  |  |  |  |  |
|  | No | 26 | 23 | 521 | 19.825(10.134-38.784) |  |  |  |  |  |  |  |
| Tumor transfer |  |  |  |  |  | <0.0001 |  |  |  |  |  |  |
|  | No | 137 | 19 | NA | 1 |  |  |  |  |  |  |  |
|  | Yes | 22 | 21 | 405 | 25.047(12.581-49.864) |  |  |  |  |  |  |  |
| Nerve infiltration |  |  |  |  |  | 0.524 |  |  |  |  |  | 0.708 |
|  | No | 157 | 39 | NA | 1 |  |  | 131 | 17 | NA | 1 |  |
|  | Yes | 2 | 1 | 1079 | 0.530 (0.073-3.859) |  |  | 1 | 0 | NA | 20.336[0-(3.700E+11)] |  |
| Postoperative chemotherapy ^c^ |  |  |  |  |  | 0.775 |  |  |  |  |  | 0.755 |
|  | No | 48 | 13 | NA | 1 |  |  | 40 | 6 | NA | 1 |  |
|  | Yes | 97 | 24 | NA | 1.103 (0.562-2.168) |  |  | 80 | 10 | NA | 1.175(0.427-3.233) |  |
|  | Missing | 14 |  |  |  |  |  | 12 |  |  |  |  |
| *ULBP2* |  |  |  |  |  | 0.047 |  |  |  |  |  | 0.032 |
|  | Negative | 79 | 15 | NA | 1 |  |  | 66 | 4 | NA | 1 |  |
|  | Positive | 80 | 25 | NA | 2.009(1.009-4.001) |  |  | 66 | 13 | NA | 9.521 (1.217-74.469) |  |

Notes: * 159 of the 161 COAD patients had information on overall survival; # 133 COAD patients underwent radical resection, of which 132 had recurrence-free survival information; a, 6 COAD patients could not obtain Tumor type information for OS and RFS; b, 4 COAD patients could not obtain tumor size information for COAD OS and RFS; c, 14 COAD patients with OS and 12 COAD patients with RFS could not obtain postoperative chemotherapy information; COAD, colon adenocarcinoma; OS, overall survival; RFS, recurrence-free survival; NA, not obtained; MST, median total survival time; MRT, Median relapse time; HR, hazard ratio; 95% CI, 95% confidence interval.
